# Effect of Solubilizing Group on the Antibacterial Activity of Heptamethine Cyanine Photosensitizers

**DOI:** 10.3390/pharmaceutics15010247

**Published:** 2023-01-11

**Authors:** Olga Semenova, Dmytro Kobzev, Iryna Hovor, Melad Atrash, Faina Nakonechny, Olesia Kulyk, Andrii Bazylevich, Gary Gellerman, Leonid Patsenker

**Affiliations:** 1 Department of Chemical Sciences, Ariel University, Ariel 40700, Israel; 2 Department of Chemical Engineering, Ariel University, Ariel 40700, Israel

**Keywords:** heptamethine cyanines, photosensitizers, antimicrobial photodynamic therapy, heavy-atom effect, solubilizing groups

## Abstract

Antibiotic resistance of pathogenic bacteria dictates the development of novel treatment modalities such as antimicrobial photodynamic therapy (APDT) utilizing organic dyes termed photosensitizers that exhibit a high cytotoxicity upon light irradiation. Most of the clinically approved photosensitizers are porphyrins that are poorly excitable in the therapeutic near-IR spectral range. In contrast, cyanine dyes function well in the near-IR region, but their phototoxicity, in general, is very low. The introduction of iodine atoms in the cyanine molecules was recently demonstrated to greatly increase their phototoxicity. Herein, we synthesized a series of the new iodinated heptamethine cyanine dyes (**ICy7**) containing various solubilizing moieties, i.e., negatively charged carboxylic (**ICy7COOH**) and sulfonic (**ICy7SO_3_H**) groups, positively charged triphenylphosphonium (**ICy7PPh_3_**), triethylammonium (**ICy7NEt_3_**) and amino (**ICy7NH_2_**) groups, and neutral amide (**ICy7CONHPr**) group. The effect of these substituents on the photodynamic eradication of Gram-positive (*S. aureus*) and Gram-negative (*E. coli* and *P. aeruginosa*) pathogens was studied. Cyanine dyes containing the amide and triphenylphosphonium groups were found to be the most efficient for eradication of the investigated bacteria. These dyes are effective at low concentrations of 0.05 µM (33 J/cm^2^) for *S. aureus*, 50 µM (200 J/cm^2^) for *E. coli*, and 5 µM (100 J/cm^2^) for *P. aeruginosa* and considered, therefore, promising photosensitizers for APDT applications. The innovation of the new photosensitizers consisted of a combination of the heavy-atom effect that increases singlet oxygen generation with the solubilizing group’s effect improving cell uptake, and with effective near-IR excitation. Such a combination helped to noticeably increase the APDT efficacy and should pave the way for the development of more advanced photosensitizers for clinical use.

## 1. Introduction

Photodynamic therapy (PDT) [1] and photodynamic antimicrobial therapy (APDT) [2] are of great importance for treating cancer [3,4,5] and eradication of pathogenic bacteria [6], viruses [7], and fungi [8] due to their non-invasiveness and minimal side effects on normal tissues. Both PDT and APDT consist of the administering of an organic dye [9] or nanoparticle [10,11] referred to as a photosensitizer (PS) [12], followed by its light-irradiation at a specific wavelength to generate reactive species that kill cells in the vicinity. As a result of the light absorption, PS molecules pass to the first excited singlet state and then turn into the long-lived triplet state, which is responsible for the photochemical reactions yielding cytotoxic species such as singlet oxygen (^1^O_2_), superoxide anion (O_2_^−•^), hydroxyl radical (^•^OH), hydrogen peroxide (H_2_O_2_), and organic radicals among others [1]. Thus, the efficient population of the triplet state plays an important role in the PS phototoxicity.

Most of the PSs approved by the Food and Drug Administration (FDA) are porphyrin-based dyes that predominantly absorb light in the short wavelength range, but insufficiently absorb in the biologically transparent and, thus, therapeutically important red and near-IR (NIR) spectral region [13,14]. In recent years, special attention is paid, therefore, to non-porphyrin-based PSs such as phthalocyanines [15,16,17] and cyanines [18,19,20] that exhibit high extinction coefficients within the NIR region. Among them, indocyanine green (**ICG**), the clinically approved NIR dye, has been widely explored as a potential photosensitizer in clinical applications [21,22]. The low phototoxicity of cyanine dyes including **ICG** is attractive for their applications as fluorescent biomedical reporters. However, a major limitation for their therapeutic utility in PDT and APDT is their unsatisfactory phototoxic effect.

The well-known approach to improve the phototoxicity of cyanine dyes is based on the introduction of heavy atoms such as iodine [23,24,25], which significantly increases the probability of intersystem crossing (ISC) from the singlet to the triplet state due to enhanced spin–orbit coupling resulting in the elevated rates of cytotoxic species generation [26]. The enhancement of PDT and APDT efficacy owing to the “heavy atom effect” has been previously reported in the example of cyanine dyes [27,28,29] among the photosensitizers of other dye classes [30,31].

**Figure 1:**
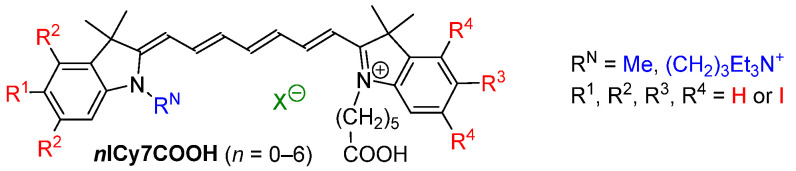
Heptamethine cyanine dyes ***n*ICy7COOH**, where *n* is the number of iodine atoms (*n* = 0–6), investigated in [32].

Recently, we reported for the first time on the unexpected effect of iodine atoms in the heptamethine cyanine dyes (***n*ICy7COOH**, where *n* = 0–6, Figure 1) on the photodynamic eradication of pathogenic bacteria, namely, *Staphylococcus aureus (S. aureus)*, *Escherichia coli (E. coli)* and *Pseudomonas aeruginosa (P. aeruginosa)*: the increasing number of iodines may not only increase but also decrease phototoxicity [32]. Thus, the iodinated cyanines containing 2–4 iodine atoms exhibited the most pronounced photo-eradication efficacy on *S. aureus*, while mono- and, surprisingly, hexa-iodinated dyes were less effective. In contrast, the mono-iodinated cyanine turned out to be the most active against *E. coli* and *P. aeruginosa*, while the phototoxicity dropped down with the increasing number of iodines. This phenomenon was attributed to the intensification of the dye aggregation in aqueous media and, thereby, the reduction in the dye uptake by these bacteria [32]. As such, an optimal balance of hydrophobic–hydrophilic properties and the selection of the more appropriate solubilizing groups are required to enhance the uptake and phototoxicity of the dyes.

A known method to improve the uptake of organic molecules (e.g., drugs and dyes) and nanoparticles by cells consists of the introduction of positively charged groups, which facilitate penetration through the negatively charged cell membrane [33,34]. Our recent results showed, however, that this is not always true: the introduction of the positively charged triethylammonium group in the di-iodinated cyanine (Figure 1) significantly improved photo-eradication of *E. coli* and *P. aeruginosa*, while unpredictably reducing the killing effect towards *S. aureus* [32].

**Figure 2:**
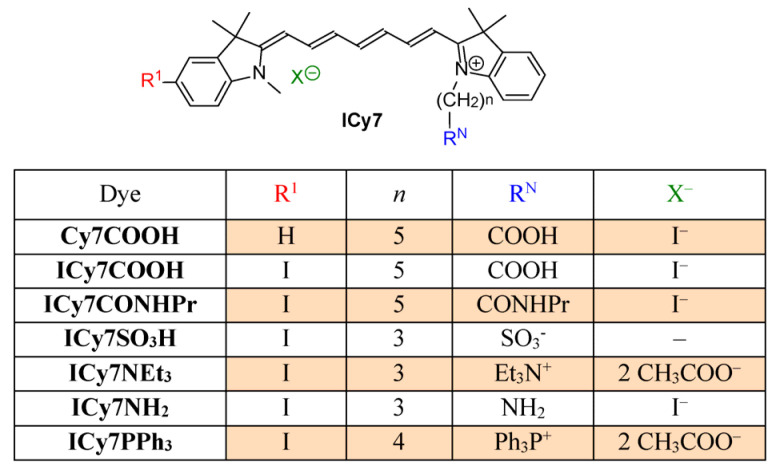
Heptamethine cyanine dyes of **ICy7** series obtained and studied in this work.

As a continuation of our recent research, this work investigated the effect of various neutrally, positively, and negatively charged solubilizing groups on the photo-eradication of *S. aureus*, *E. coli*, and *P. aeruginosa*. To this end, we synthesized a series of novel iodinated heptamethine cyanine dyes containing carboxylic (**ICy7COOH**), amide (**ICy7CONHPr**), sulfonic (**ICy7SO_3_H**), triphenylphosphonium (**ICy7PPh_3_**), triethylammonium (**ICy7NEt_3_**), and amino (**ICy7NH_2_**) groups (Figure 2). We studied their photophysical properties, uptake by bacteria, and photodynamic efficacy in comparison with the non-iodinated cyanine, **Cy7COOH**. To minimize the unwanted effects associated with possible dye aggregation, our investigation was performed using the example of the mono-iodinated cyanines, **ICy7**.

## 2. Materials and Methods

### 2.1. General

All starting materials and chemicals were supplied by Alfa Aesar and Sigma Aldrich. Solvents were purchased from Bio-Lab Israel.

Chemical reactions were monitored by TLC (Silica gel 60 F-254, Merck) and LCMS. LCMS analyses were performed using an Agilent Technologies 1260 Infinity (LC) 6120 quadruple (MS) instrument, column Agilent SB-C18, 1.8 mm, 2.1 × 50 mm, column temperature 50 °C, eluent water–acetonitrile (ACN) + 0.1% formic acid. HRMS analyses were performed in ESI positive mode by using an Agilent 6550 iFunnel Q-TOF LCMS instrument. ^1^H NMR and ^13^C NMR spectra were measured in DMSO-*d*_6,_ CD_3_OD, and a mixture CDCl_3_–CD_3_OD on a Bruker Avance III HD (400 MHz and 100 MHz for ^1^H and ^13^C, respectively) spectrometer. Chemical shifts were reported in ppm using the solvent residual signal as an internal reference: δ_H_(DMSO-*d*_6_) = 2.50 ppm, δ_C_(DMSO-*d*_6_) = 39.52 ppm; δ_H_(CDCl_3_) = 7.26 ppm, δ_C_(CDCl_3_) = 77.16 ppm; δ_H_(CD_3_OD) = 3.31 ppm, δ_C_(CD_3_OD) = 49.0 ppm.

### 2.2. Synthesis

**1,2,3,3-Tetramethyl-3*H*-indol-1-ium (1a) and 5-iodo-1,2,3,3-tetramethyl-3*H*-indol-1-ium (1b)** were synthesized according to the procedure described in [35,36]: 2,3,3-trimethyl-3*H*-indole (1 g, 6.28 mmol) or 5-iodo-2,3,3-trimethyl-3*H*-indole (1 g, 3.51 mmol) was refluxed overnight with excess of methyl iodide (10 eq.). The precipitate was washed with diethyl ether and dried in a desiccator under vacuum.

**1,2,3,3-Tetramethyl-3*H*-indol-1-ium (1a).** Yield: 1.61 g (85%). ^1^H NMR (400 MHz, DMSO-*d_6_*, ppm): δ 7.91 (1H, d, *J* = 5.2 Hz), 7.83 (1H, d, *J* = 5.4 Hz), 7.62 (2H, t, *J* = 5.6 Hz), 3.98 (3H, s), 2.77 (3H, s), 1.53 (6H, s). MS *m*/*z* C_12_H_16_N^+^ calculated [M]^+^ 174.13, found *m*/*z* 174.10.

**5-Iodo-1,2,3,3-tetramethyl-3*H*-indol-1-ium (1b):** Yield: 0.96 g (65%). ^1^H NMR (400 MHz, DMSO-*d_6_*, ppm): δ 8.27 (1H, s), 7.99 (1H, d, *J* = 8.5 Hz), 7.71 (1H, d, *J* = 8.4 Hz), 3.93 (3H, s), 2.73 (3H, s), 1.51 (6H, s). MS *m*/*z* C_12_H_15_IN^+^ calculated [M]^+^ 300.02, found *m*/*z* 300.16.

**1-(5-Carboxypentyl)-2,3,3-trimethyl-3*H*-indol-1-ium bromide (3a)** was synthesized as described in [32]. 2,3,3-Trimethyl-3*H*-indole (1.0 g, 6.28 mmol) was mixed with 6-bromohexanoic acid (1.84 g, 9.41 mmol) and heated at 120 °C for 15 h in a sealed tube. The reaction mixture was cooled to r.t., then diluted with benzene (5 mL); then, the solvent was decanted, and the residue was triturated with benzene (3 × 5 mL), and filtered. The obtained precipitate was washed with acetone (1 mL) and dried to yield **3a** (1.33 g, 60%). ^1^H NMR (400 MHz, DMSO-*d_6_*, ppm): δ 7.97 (1H, d, *J* = 5.1 Hz), 7.84 (1H, d, *J* = 5.4 Hz), 7.62 (2H, t, *J* = 5.1 Hz), 4.45 (2H, t, *J* = 7.8 Hz), 2.84 (3H, s), 2.23 (2H, t, *J* = 7.2 Hz), 1.85 (2H, m), 1.56 (2H, m), 1.54 (6H, s), 1.43 (2H, m). MS *m*/*z* C_17_H_24_NO_2_^+^ calculated [M]^+^ 274.18, found *m*/*z* 274.20.

**3-(2,3,3-Trimethyl-3*H*-indol-1-ium-1-yl)propane-1-sulfonate (3b)** was synthesized similarly to [37]. 2,3,3-trimethyl-3H-indole (1.5 g, 9.4 mmol) and 1,3-propanesultone (2.8 g, 22.9 mmol) were melted, at 120 °C, for 2 h. The reaction mixture became solid. 2-Propanol (5 mL) was added to the reaction mixture and heated, at 80 °C, for 1 h. Then, the reaction mixture was washed with diethyl ether (30 mL) and dried in a desiccator under vacuum. Then, the obtained viscous liquid was diluted with H_2_O and column purified on LiChroprep RP18 using 0–1% acetonitrile–water as eluent resulting in **3b** (light-yellow viscous liquid). Yield: 68%. ^1^H NMR (400 MHz, DMSO-*d_6_*, ppm): δ 8.04 (1H, d, *J* = 6.9 Hz), 7.82 (1H, d, *J* = 8.0 Hz), 7.69–7.56 (2H, m), 4.65 (2H, t, *J* = 8.0 Hz), 2.83 (3H, s), 2.64 (2H, t, *J* = 6.5 Hz), 1.53 (6H, s). MS *m*/*z* C_14_H_19_NO_3_S calculated [M + H]^+^ 281.11, found *m*/*z* 282.21.

**2,3,3-Trimethyl-1-(4-(triphenylphosphonio)butyl)-3*H*-indol-1-ium dibromide (3c)** was synthesized as described in [38]. A mixture of 2,3,3-trimethylindolenine (500 mg, 3.14 mmol), 4-bromobutyltriphenylphosphonium bromide (4.5 g, 9.42 mmol) and potassium iodide (1.56 g, 9.42 mmol) in acetonitrile was refluxed for 72 h. After cooling to r.t., water (50 mL) and ethyl acetate (50 mL) were added to the reaction mixture, and the organic layer was separated. The aqueous phase containing the product was concentrated under reduced pressure, and the residue was washed three times with diethyl ether and dried in a desiccator under vacuum to give **3c**. Yield: 74%. ^1^H NMR (400 MHz, DMSO-*d_6_*, ppm): δ 8.05–7.98 (1H, m), 7.94–7.71 (16H, m), 7.64–7.57 (2H, m), 4.56 (2H, t, *J* = 7.4 Hz), 3.86–3.71 (2H, m), 2.84 (3H, s), 2.16–2.05 (2H, m), 1.78–1.63 (2H, m), 1.48 (6H, s). MS *m*/*z* C_33_H_36_NP^2+^ calculated [M]^2+^ 238.63, found *m*/*z* 238.60.

**2,3,3-Trimethyl-1-(3-(triethylammonio)propyl)-3*H*-indol-1-ium dibromide (3d)** was synthesized similar to [32]. 2,3,3-trimethyl-3H-indole (0.5 g, 3.1 mmol) and 3-bromo-N,N,N-triethylpropan-1-aminium bromide (0.95 g, 3.1 mmol) were stirred in acetonitrile (14 mL) at reflux for 4 days. The reaction mixture was precipitated with diethyl ether (30 mL). Then, the solvent was decanted and the viscous solid was triturated with diethyl ether, filtered, washed with ether, and dried in a desiccator under vacuum to give **3d**. Yield: (0.6 g) 62%. ^1^H NMR (400 MHz, DMSO-*d_6_*, ppm): δ 8.13 (1H, d, *J* = 7.3 Hz), 7.86 (1H, d, *J* = 7.8 Hz), 7.71–7.59 (2H, m), 4.58 (2H, t, *J* = 8.0 Hz), 3.33–3.18 (8H, m), 2.93 (3H, s), 2.29–2.13 (2H, m), 1.56 (6H, s), 1.23 (9H, t, *J* = 7.1 Hz). MS *m*/*z* C_20_H_34_N_2_^2+^ calculated [M]^2+^ 151.13, found *m*/*z* 151.20.

**1-(3-Aminopropyl)-2,3,3-trimethyl-3*H*-indol-1-ium bromide (3e)** was synthesized as described in [39]: 2,3,3-trimethylindolenine (0.5 g, 3.14 mmol), 3-bromopropylamine hydrobromide (1.87 g, 8.61 mmol) and anhydrous toluene (10 mL) were added to a sealed tube. The suspension was heated, at 130 °C, for 4 days. After cooling to r.t., toluene was decanted. The residue was washed with toluene and triturated with acetonitrile, filtered, washed with diethyl ether, and dried to give **3e.** Yield: 0.31 g (50%). ^1^H NMR (400 MHz, DMSO-*d_6_*, ppm): δ 8.06 (1H, d, *J* = 8.0 Hz), 7.98 (2H, br. s), 7.85 (1H, d, *J* = 7.9 Hz), 7.68–7.59 (2H, m), 4.59 (2H, t, *J* = 8.0 Hz), 3.12–3.00 (2H, m), 2.88 (3H, s), 2.17 (2H, t, *J* = 7.9 Hz), 1.56 (6H, s). MS *m*/*z* C_14_H_21_N_2_^+^ calculated [M]^+^ 217.17, found *m*/*z* 217.20.

**General procedure for the synthesis of the dyes Cy7COOH, ICy7COOH ICy7SO_3_H, ICy7NEt_3_ and ICy7PPh_3_.**

A solution of indolenine **1a** or **1b** (0.5 mmol) and *N*-[5-(phenylamino)-2,4-pentadienylidene]aniline hydrochloride (0.5 mmol) in acetic anhydride (2 mL) was heated, at 90 °C, for 20 min to form the corresponding intermediate product **2a** or **2b**. The reaction mixture was cooled to r.t., and a solution of the second indolenine **3a**–**3d** (0.5 mmol) in dry pyridine (2 mL) was added. The reaction mixture was heated, at 90 °C, for 10 min, and the obtained cyanine dye was precipitated with diethyl ether, filtered, and washed with diethyl ether.

**6-(3,3-Dimethyl-2-(7-(1,3,3-trimethylindolin-2-ylidene)hepta-1,3,5-trien-1-yl)-3*H*-indol-1-ium-1-yl)hexanoate (Cy7COOH)** was column purified on Silica gel 60 using 10% methanol–chloroform as eluent. Yield: 119 mg (52%). ^1^H NMR (400 MHz, CDCl_3_–CD_3_OD, ppm): δ 7.75 (2H, t, *J* = 13.1 Hz, CH), 7.46 (1H, t, *J* = 12.7 Hz, CH), 7.40–7.34 (2H, m, ArH), 7.36 (2H, d, *J* = 7.5 Hz, ArH), 7.22 (2H, t, *J* = 7.4 Hz, ArH), 7.12 (1H, d, *J* = 8.4 Hz, ArH), 7.09 (1H, d, *J* = 8.4 Hz, ArH), 6.51 (2H, t, *J* = 12.5 Hz, CH), 6.11 (1H, d, *J* = 13.4 Hz, CH), 6.09 (1H, d, *J* = 13.6 Hz, CH), 3.96 (2H, t, *J* = 7.4 Hz, NCH_2_), 3.57 (3H, s, NCH_3_), 2.31 (2H, t, *J* = 7.2 Hz, CH_2_COOH), 1.86–1.72 (2H, m, CH_2_), 1.65 (12H, s, 2 (CH_3_)_2_), 1.74–1.62 (2H, m, CH_2_), 1.53–1.41 (2H, m, CH_2_).^13^C NMR (100 MHz, CDCl_3_–CD_3_OD, ppm): δ 175.63, 171.14, 170.56, 155.75, 150.53, 141.67, 141.00, 139.80, 139.63, 127.84, 124.76, 124.26, 121.36, 121.23, 109.50, 109.40, 102.68, 102.42, 48.47_,_ 43.09, 32.98, 30.23, 26.83, 26.19, 25.45, 23.58. HRMS *m*/*z* (ESI^+^) C_34_H_40_N_2_O_2_^+^ calculated [M]^+^ 509.3168, found *m*/*z*: 509.3162.

**6-(2-(7-(5-Iodo-1,3,3-trimethylindolin-2-ylidene)hepta-1,3,5-trien-1-yl)-3,3-dimethyl-3*H*-indol-1-ium-1-yl)hexanoate (ICy7COOH)** was column purified on Silica gel 60 using 5–10% methanol–chloroform as eluent. Yield: 74 mg (45%). ^1^H NMR (400 MHz, CDCl_3_–CD_3_OD, ppm): δ 7.79 (1H, t, *J* = 13.1 Hz, CH), 7.63 (1H, t, *J* = 13.1 Hz, CH), 7.62 (1H, d, *J* = 8.5 Hz, ArH), 7.57 (1H, s, ArH), 7.43 (1H, t, *J* = 12.7 Hz, CH), 7.39 (1H, t, *J* = 7.9 Hz, ArH), 7.38 (1H, d, *J* = 7.8 Hz, ArH), 7.28 (1H, t, *J* = 7.4 Hz, ArH), 7.18 (1H, d, *J* = 7.9 Hz, ArH), 6.80 (1H, d, *J* = 8.4 Hz, ArH), 6.56 (1H, t, *J* = 13.3 Hz, CH), 6.47 (1H, t, *J* = 13.3 Hz, CH), 6.23 (1H, d, *J* = 13.5 Hz, CH), 5.95 (1H, d, *J* = 13.3 Hz, CH), 4.02 (2H, t, *J* = 7.4 Hz, NCH_2_), 3.45 (3H, s, NCH_3_), 2.27 (2H, t, *J* = 7.2 Hz, CH_2_COOH), 1.86–1.74 (2H, m, CH_2_), 1.72–1.63 (2H, m, CH_2_), 1.66 (6H, s, 2 CH_3_), 1.62 (6H, s, 2 CH_3_), 1.52–1.42 (2H, m, CH_2_). ^13^C NMR (100 MHz, CDCl_3_–CD_3_OD, ppm): δ 176.41, 172.49, 168.14, 155.64, 152.05, 148.66, 141.84, 141.47, 140.63, 140.16, 136.45, 130.15, 128.07, 125.47, 125.20, 125.02, 121.42, 110.53, 110.27, 104.15, 101.49, 86.20, 48.73, 47.34_,_ 43.55, 33.74, 29.95, 26.86, 26.68, 26.36, 25.42, 23.80. HRMS *m*/*z* (ESI^+^) C_34_H_39_IN_2_O_2_^+^ calculated [M]^+^ 635.2134, found *m*/*z*: 635.2127.

**3-(2,7,5-Iodo-1,3,3-trimethylindolin-2-ylidene)hepta-1,3,5-trien-1-yl)-3,3-dimethyl-3*H*-indol-1-ium-1-yl)propane-1-sulfonate (ICy7SO_3_H)** was column purified on Silica gel 60 using 2–12% methanol–chloroform as eluent. Yield: 18%. ^1^H NMR (400 MHz, CD_3_OD, ppm): δ 7.96 (1H, t, *J* = 12.9 Hz, CH), 7.80 (1H, t, *J* = 13.2 Hz, CH), 7.74 (1H, s, ArH), 7.66 (1H, d, *J* = 8.3, ArH), 7.58–7.49 (2H, m, ArH, CH), 7.49–7.38 (2H, m, ArH), 7.28 (1H, t, *J* = 7.2 Hz, ArH), 7.02 (1H, d, *J* = 8.3 Hz, ArH), 6.59 (1H, t, *J* = 12.7 Hz, CH), 6.52 (1H, d, *J* = 13.3 Hz, CH), 6.50 (1H, t, *J* = 12.9 Hz, CH), 6.12 (1H, d, *J* = 13.4 Hz, CH), 4.37 (2H, t, NCH_2_), 3.51 (3H, s, NCH_3_), 3.00 (2H, t, *J* = 6.8 Hz, CH_2_SO_3_^−^), 2.30–2.18 (2H, m, CH_2_), 1.67 (6H, s, 2 CH_3_), 1.64 (6H, s, 2 CH_3_). ^13^C NMR (100 MHz, CD_3_OD, ppm): δ 174.86, 170.85, 157.78, 154.66, 151.43, 144.57, 144.36, 143.31, 142.76, 138.56, 132.31, 129.98, 128.02, 127.47, 126.77, 123.50, 113.04, 112.57, 106.59, 103.97, 87.69, 79.55, 54.90, 50.80, 44.05, 31.09, 27.85, 27.76, 24.27. HRMS: *m*/*z* (ESI+) C_31_H_36_IN_2_O_3_S calculated [M+H]^+^ 643.1491, found *m*/*z*: 643.1492.

**2,7,5-Iodo-1,3,3-trimethylindolin-2-ylidene)hepta-1,3,5-trien-1-yl)-3,3-dimethyl-1-(3-(triethylammonio)propyl)-3*H*-indol-1-ium (ICy7NEt_3_)** was column purified on LiChroprep RP18 using 5–50% acetonitrile–water + 0.05% acetic acid as eluent. Yield: 38%. ^1^H NMR (400 MHz, CD_3_OD, ppm): δ 8.05–7.91 (2H, m, CH), 7.86 (1H, s, ArH), 7.75 (1H, d, *J* = 8.3 Hz, ArH), 7.64 (1H, t, *J* = 12.8 Hz, CH), 7.50 (1H, d, *J* = 7.4 Hz, ArH), 7.42 (1H, t, *J* = 7.6 Hz, ArH), 7.36 (1H, d, *J* = 8.0 Hz, ArH), 7.24 (1H, t, *J* = 7.4 Hz, ArH), 7.14 (1H, d, *J* = 8.4 Hz, ArH), 6.70–6.55 (m, 2H, CH), 6.40 (1H, d, *J* = 13.4 Hz, CH), 6.35 (1H, d, *J* = 13.7 Hz, CH), 4.21 (2H, t, *J* = 7.4 Hz, NCH_2_), 3.61 (3H, s, NCH_3_), 3.36 (8H, q, *J* = 6.9 Hz, N(CH_2_)_4_), 2.24–2.11 (m, 2H, CH_2_), 1.91 (6H, s, 2 CH_3_COO^−^), 1.71 (6H, s, 2 CH_3_), 1.69 (6H, s, 2 CH_3_), 1.31 (9H, t, *J* = 7.2 Hz, 3 CH_3_). ^13^C NMR (100 MHz, CD_3_OD, ppm): δ 179.45, 174.00, 172.06, 158.22, 154.11, 152.76, 144.86, 144.16, 143.48, 142.13, 138.80, 132.56, 129.91, 127.94, 127.72, 125.87, 123.63, 114.00, 111.28, 106.01, 104.45, 89.53, 79.55, 54.90, 54.77, 54.05, 50.42, 50.10, 49.00, 41.20, 31.67, 28.08, 27.56, 23.59, 20.95, 7.71. HRMS: *m*/*z* (ESI+) C_37_H_50_IN_3_^2+^ calculated [M]^2+^ 331.6519, found *m*/*z*: 331.6523.

**2,7,5-Iodo-1,3,3-trimethylindolin-2-ylidene)hepta-1,3,5-trien-1-yl)-3,3-dimethyl-1-(4-(triphenylphosphonio)butyl)-3*H*-indol-1-ium (ICy7PPh_3_)** was column purified on LiChroprep RP18 using 0–60% acetonitrile–water + 0.5% acetic acid as eluent. Yield: 34%. ^1^H NMR (400 MHz, CD_3_OD, ppm): 7.93–7.84 (4H, m, 2 ArH, 2 CH), 7.82–7.67 (15H, m, 3 Ph), 7.62 (1H, t, *J* = 12.9 Hz, CH), 7.47 (1H, d, *J* = 7.4 Hz, ArH), 7.42–7.32 (2H, m, ArH), 7.25 (1H, t, *J* = 7.1 Hz, ArH), 7.07 (1H, d, *J* = 8.4 Hz, ArH), 6.67–6.52 (2H, m, 2 CH), 6.46 (1H, d, *J* = 13.6 Hz, CH), 6.23 (1H, d, *J* = 13.5 Hz, CH), 4.26 (2H, t, *J* = 7.0 Hz, NCH_2_), 3.61–3.46 (2H, m, CH_2_P), 3.56 (3H, s, NCH_3_), 2.17–2.06 (2H, m, CH_2_), 1.95 (6H, s, 2 CH_3_COO^−^), 1.84–1.72 (2H, m, CH_2_), 1.66 (6H, s, 2 CH_3_), 1.56 (6H, s, 2 CH_3_). ^13^C NMR (100 MHz, CD_3_OD, ppm): 177.42, 174.09, 171.95, 157.94, 153.93, 152.39, 144.54, 144.41, 143.31, 142.54, 138.64, 136.34, 134.93, 134.83, 132.41, 131.66, 131.53, 129.84, 127.67, 127.44, 126.44, 123.53, 120.03, 119.17, 113.38, 112.43, 106.07, 104.64, 88.36, 50.49, 44.21, 31.47, 29.63, 29.45, 27.92, 27.82, 22.50, 22.27, 21.98, 21.31. HRMS: *m*/*z* (ESI^+^) C_50_H_52_IN_2_P^2+^ calculated [M]^+^ 838.2913, found *m*/*z*: 838.2936.

**Synthesis of 2,7,5-iodo-1,3,3-trimethylindolin-2-ylidene)hepta-1,3,5-trien-1-yl)-3,3-dimethyl-1-(6-oxo-6-(propylamino)hexyl)-3H-indol-1-ium iodide (ICy7CONHPr).**

A mixture of **ICy7COOH** (50 mg, 0.066 mmol), *N*,*N*,*N′*,*N′*-tetramethyl-*O*-(*N*-succinimidyl)uronium tetrafluoroborate (TSTU) (39.7 mg, 0.132 mmol) and *N*,*N*-diisopropylethylamine (DIPEA) (46 μL, 0.264 mmol) was dissolved in dry DMF (0.5 mL). The reaction mixture was stirred, at r.t., for 40 min and then propylamine (21 μL, 0.264 mmol) was added. After stirring, at r.t., for 30 min the obtained dye was precipitated with diethyl ether, filtered off and washed with diethyl ether. The raw product was column purified on Silica gel 60 using 5–10% methanol–chloroform as eluent. Yield: 33 mg (62%). ^1^H NMR (400 MHz, CD_3_OD, ppm): δ 8.02 (1H, t, *J* = 13.2 Hz, CH), 7.85 (1H, t, *J* = 13.2 Hz, CH), 7.74 (1H, s, ArH), 7.68 (1H, d, *J* = 8.9 Hz, ArH), 7.63 (1H, t, *J* = 12.7 Hz, CH), 7.52 (1H, d, *J* = 7.3 Hz, ArH), 7.44 (1H, t, *J* = 7.9 Hz, ArH), 7.36 (1H, d, *J* = 7.9 Hz, ArH), 7.31 (1H, t, *J* = 7.4 Hz, ArH), 6.99 (1H, d, *J* = 8.4 Hz, ArH), 6.62 (1H, t, *J* = 13.3 Hz, CH), 6.54 (1H, t, *J* = 13.3 Hz, CH), 6.43 (1H, d, *J* = 13.8 Hz, CH), 6.12 (1H, d, *J* = 13.2 Hz, CH), 4.17 (2H, t, *J* = 7.4 Hz, NCH_2_), 3.49 (3H, s, NCH_3_), 3.08 (2H, t, *J* = 7.4 Hz, CH_2_CONH), 2.20 (2H, t, *J* = 7.2 Hz, CH_2_CONH), 1.88–1.76 (2H, m, CH_2_), 1.74–1.60 (2H, m, CH_2_), 1.71 (6H, s, 2 CH_3_), 1.66 (6H, s, 2 CH_3_), 1.53–1.39 (4H, m, 2 CH_2_), 0.88 (3H, t, *J* = 7.4 Hz, CH_3_). Amino group (-NH-) has an exchangeable proton which is not recorded in ^1^H NMR spectrum due to the low concentration. ^13^C NMR (100 MHz, CD_3_OD, ppm): δ 175.73, 175.27, 170.96, 157.72, 154.56, 151.33, 144.61, 144.34, 143.40, 142.94, 138.59, 132.32, 129.92, 127.62, 127.68, 126.90, 123.53, 112.89, 112.66, 106.50, 103.65, 87.54, 52.93, 49.56, 49.28, 45.26, 42.17, 36.66, 31.13, 30.75, 28.44, 27.98, 27.83, 27.31, 26.55, 23.64, 11.76. HRMS *m*/*z* (ESI^+^) C_37_H_47_IN_3_O^+^ calculated [M]^+^ 676.2764, found *m*/*z*: 676.2789.

**Synthesis of 1-(3-aminopropyl)-2,7,5-iodo-1,3,3-trimethylindolin-2-ylidene)hepta-1,3,5-trien-1-yl)-3,3-dimethyl-3H-indol-1-ium iodide (ICy7NH_2_).** A mixture of indolenine **1b** (200 mg, 0.47 mmol) and *N*-[5-(phenylamino)-2,4-pentadienylidene]aniline hydrochloride (133 mg, 0.47 mmol) was dissolved in acetic anhydride (2 mL) and heated, at 90 °C, for 15 min to form product **2b**. Then, the solvent was evaporated to dryness, the residue **2b** was dried in a desiccator under vacuum for 3 h. The solution of the second indolenine **3e** (139 mg, 0.47 mmol) in DMF (2 mL) and pyridine (200 µL) was added to the product **2b**. The reaction mixture was heated, at 90 °C, for 5 min, and cooled to r.t.; the obtained product was precipitated with diethyl ether, filtered, and washed with diethyl ether. The raw dye was column purified on Silica gel 60 using 5–18% methanol–chloroform as eluent. Yield: 37 mg (15%). ^1^H NMR (400 MHz, CDCl_3_–CD_3_OD, ppm): δ 7.88 (1H, t, *J* = 13.2 Hz, CH), 7.82 (1H, t, *J* = 13.2 Hz, CH), 7.69 (1H, d, *J* = 8.5 Hz, ArH), 7.68 (1H, s, ArH), 7.55 (1H, t, *J* = 12.9 Hz, CH), 7.46–7.36 (2H, m, ArH), 7.30 (1H, d, *J* = 8.2 Hz, ArH), 7.24 (1H, t, *J* = 7.3 Hz, ArH), 6.96 (1H, d, *J* = 7.8 Hz, ArH), 6.79 (1H, t, *J* = 13.2 Hz, CH), 6.58 (1H, t, *J* = 12.5 Hz, CH), 6.49 (1H, d, *J* = 13.5 Hz, CH), 6.15 (1H, d, *J* = 13.5 Hz, CH), 4.21 (2H, t, *J* = 9.1 Hz, NCH_2_), 3.54 (3H, s, NCH_3_), 3.20 (2H, t, *J* = 7.8 Hz, CH_2_), 2.24–2.10 (2H, m, CH_2_), 1.69 (6H, s, 2 CH_3_), 1.67 (6H, s, 2 CH_3_). Amino group (NH_2_) has exchangeable protons which are not recorded in ^1^H NMR spectrum due to the low concentration. ^13^C NMR (100 MHz, CDCl_3_–CD_3_OD, ppm): δ 151.53, 143.43, 142.31, 141.57, 138.14, 131.78, 131.72, 130.36, 129.49, 126.08, 122.91, 112.59, 111.42, 105.33, 104.08, 88.26, 49.50, 49.28, 42.00, 37.72, 31.43, 30.14, 29.77, 28.17, 28.06, 25.73. HRMS *m*/*z* (ESI^+^) C_31_H_37_IN_3_ calculated [M]^+^ 578.2032, found *m*/*z*: 578.2033.

### 2.3. Absorption and Fluorescence Measurements

The absorption spectra were recorded on a Jasco V-730 UV-Vis spectrophotometer and the fluorescence spectra were taken on an Edinburgh FS5 spectrofluorometer. The absorption and fluorescence spectra were measured, at 25 °C, in standard 1 cm quartz cells at ~1 µM dye concentrations in MeOH and 0.7% DMSO in aqueous saline. The excitation wavelength (λ*) was 680 nm (in methanol) and 700 nm (in aqueous saline).

To determine the fluorescence quantum yields (Φ_F_), the integrated relative intensities of the dyes were measured vs. the commercially available disulfonated **Cy7** (SETA BioMedicals, https://www.setabiomedicals.com) in phosphate buffer pH 7.4 as the reference (Φ_F_ = 13% [40]); in addition, the quantum yields were calculated according to Equation (1) [41].

Φ_F_ = Φ_FRef_ × (*F*/*F*_Ref_) × (*A*_Ref_/*A*) × (*n_D_*_(*media*)_^2^/*n_D_*_(*Ref*)_^2^), (1)

where Φ_FRef_ is the quantum yield of the reference, *F*_Ref_ and *F* are the areas (integral intensities) of the emission spectra (*F* = ∫*I(*λ*)d*λ) of the reference dye and the dye under examination, *A*_Ref_ and *A,* are the absorbances at the excitation wavelength of the reference dye and the dye under examination, and *n_D(Ref)_* and *n_D(media)_* are the refractive indices of the solvents used for the reference dye and the dye under examination, respectively.

The extinction coefficient and the quantum yield for each dye were independently measured three times, and the average value was taken.

### 2.4. Quantum Yield of Singlet Oxygen Generation

The quantum yields of the singlet oxygen generation (Φ_Δ_) in *methanol* were measured according to the procedure [42]. Solutions containing (i) 1,3-diphenylisobenzofuran (**DPBF**, *A*~0.3, *c*~1.3 × 10^−5^ M) as the singlet oxygen scavenger and (ii) a dye under investigation or reference dye (*A*~1.0, *c*~4−5 × 10^−6^ M) in methanol were prepared. 1,1′,3,3,3′,3′-Hexamethylindotricarbocyanine iodide (**HITC**) was used as the reference dye. The obtained solutions (3.0 mL) were light irradiated in a standard 1 cm quartz cells by using a 730 nm, 30 W LED equipped with a 60° lens from the distance of 55 cm, and the absorption spectra were recorded over time. The total irradiation time was in the range of 20–120 min. During this time, the absorbance of **DPBF** reduced to about 10% of its initial value. The corresponding plot representing the absorbance of **DPBF** at 410 nm vs. time was drawn and fitted by a first-order reaction rate function. Then, the singlet oxygen generation quantum yield (Φ_Δ_) was calculated relative to the reference dye (**HITC**) (Φ_ΔRef_ = 0.89% [42]) according to Equation (2).

Φ_Δ_ = Φ_ΔRef_ × (*r*/*r*_Ref_) × (*A*_Ref_/*A*), (2)

where Φ_ΔRef_ is the quantum yield of the singlet oxygen generation for the reference dye, *r*_Ref_ and *r* are the rates of singlet oxygen scavenger degradation obtained from the corresponding fitting curves of the reference dye and the dye under examination, and *A*_Ref_ and *A* are the absorbances at λ* = 730 nm of the reference dye and the dye under examination.

The quantum yields of the singlet oxygen generation (Φ_Δ_) in *aqueous saline* were measured according to the procedure [43]. In standard 1 cm quartz cells, the solutions of Singlet Oxygen Sensor Green (**SOSG**, *c*~6 × 10^−6^ M) [44] and a dye under investigation or reference dye (*c*~1 × 10^−6^ M) in saline were prepared. Indocyanine Green (**ICG**) was used as the reference dye. The obtained solutions (3 mL) were light irradiated by a 730 nm, 30 W LED equipped with a 60° lens from the distance of 26.5 cm and the emission spectra were recorded over time. The total irradiation time was in the range of 6–30 min. During this time, the emission of **SOSG** gradually increased. The corresponding plots representing the emission of **SOSG** at 530 nm vs. time were drawn and fitted by a zero-order reaction rate function [43]. Then, the singlet oxygen generation quantum yield Φ_Δ_ was calculated relative to the reference dye (**ICG**) (Φ_ΔRef_ = 0.2% [45]) according to Equation (2) [43].

Each experiment on the Φ_Δ_ measurements in methanol and aqueous saline was carried out in triplicate, and the average Φ_Δ_ was taken. The reproducibility in the determination of Φ_Δ_ was no worse than 5%.

### 2.5. Dye Uptake by Cells

Suspensions of *S. aureus* and *E. coli* in saline (10^3^–10^4^ cells/mL, 2.5 mL) were incubated with the investigated dye (*c*_Dye_ = 1 µM) in the dark for 30 min (25 °C), and the fluorescence intensity was measured at the dye emission maximum. Then, the bacteria were isolated by centrifugation (6000 rpm for 6 min), resuspended in saline (2.5 mL), and the fluorescence spectra of the suspensions of resuspended cells and supernatant were again measured. The dye uptake was quantified as the ratio of fluorescence intensities for the suspensions of resuspended cells and supernatants. Each measurement was performed in triplicate and the average value was taken.

### 2.6. Antimicrobial Studies

Cultures of *S. aureus* (ATCC 25923), *E. coli* (ATCC 25922) and *P. aeruginosa* (ATCC 25668) were grown on Brain Heart agar plates (BHA, Acumedia, Lansing, MI, USA) for 24 h, transferred into Brain Heart broth (BH, Acumedia, Lansing, MI, USA), grown at 37 ± 1 °C with shaking at 170 rpm until reaching the absorbance *A* = 0.10 ± 0.02 at 660 nm, which corresponded to a final concentration of 10^8^ cells/mL, and the cells were diluted with commercially available sterile 0.9% saline solution to the final concentration of 10^3^–10^4^ cells/mL. This cell population was found to be appropriate for further dilutions, cell growth, and calculations, in particular, when we compare sensitizers exhibiting different phototoxicity.

All preparatory operations with photosensitizers were carried out in the dark to avoid their activation and photobleaching. The stock solutions of the dyes in DMSO (0.1–0.5 mM for *S. aureus* and 7.4–7.8 mM for *E. coli* and *P. aeruginosa* spectrophotometric control) were prepared, and the desirable final concentrations were achieved in up to three dilutions. Then, each dye solution in DMSO (7 µL) was added to bacterial suspensions (1 mL) in 0.9% saline (Falcon^®^ 24-well polystyrene clear flat bottom plate was used). Thus, the amount of DMSO added to the bacterial suspensions was always 0.7%. The bacterial suspensions were incubated in the dark, at r.t., for 30 min and then exposed to light with shaking (or kept in the dark for the control) for certain periods of time (1, 3, 10, 30, 60, and 120 min for *S. aureus* and 30, 60 and 120 min for *E. coli* and *P. aeruginosa*). The light exposure was carried out by a 730 nm, 30 W LED equipped with a 60° lens from the distance of 8 cm (light power density 56 mW/cm^2^).

After the light exposure, aliquots of each sample (100 µL) were spread over BHA plates with a Drigalsky spreader, incubated at 37 ± 1 °C for 24 h, and the colony forming units (CFU) were counted using a colony counter Scan 500 (Interscience, Saint-Nom-la-Bretèche, France).

To verify the dark toxicity of the dyes, the same experiments were carried out in parallel without light exposure. As controls we utilized the samples of bacteria (i) without dye in the dark and (ii) without dye upon light irradiation.

All the experiments with bacteria were carried out in triplicate 4–5 times in different days and the average values were taken. The results are expressed as the mean ± standard deviation (SD).

## 3. Results and Discussion

### 3.1. Overview of the Dye Structures

**Scheme 1:**
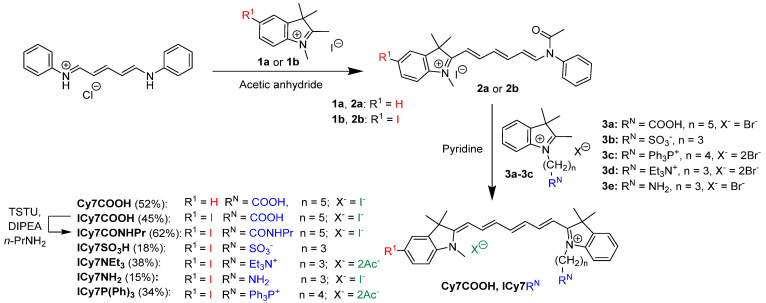
Synthesis of heptamethine cyanine dyes.

All the investigated heptamethine cyanine dyes of **ICy7** series had asymmetric structure; they contained a constant, 5-iodo-1,3,3-trimethyl indolenine at the one end, with a variable moiety at the second end—indolenine quaternized with carboxypentyl (**ICy7COOH**), N-propylamide (**ICy7CONHPr**), propanesulfonate (**ICy7SO_3_H**), butyl triphenylphosphonium (**ICy7PPh_3_**), propyl triethylammonium (**ICy7NEt_3_**) or propyl amino (**ICy7NH_2_**) group (Scheme 1). As the reference “parent” dye, we utilized the known non-iodinated cyanine, **Cy7COOH** [32]. While the carboxylic and sulfonic groups in **Cy7COOH**, **ICy7COOH** and **ICy7SO_3_H** can be deprotonated in aqueous media to provide a negative charge for these groups, amide group in **ICy7CONHPr** is neutral, amino group in **ICy7NH_2_** can be protonated, and triethylammonium (**ICy7NEt_3_**) and triphenylphosphonium (**ICy7PPh_3_**) groups bear permanent positive charge.

### 3.2. Synthesis

The heptamethine cyanine dyes were synthesized similar to [32] with reasonable yields by a one-pot sequential reaction of *N*-[5-(phenylamino)-2,4-pentadienylidene]aniline with the first indolenine molecule **1a** or **1b** in acetic anhydride to form a corresponding *N*-phenylacetamide derivative **2a** or **2b**, which was then reacted with the second indolenine **3a**–**3e** in the presence of pyridine (Scheme 1).

### 3.3. Spectral Properties and Quantum Yields of Singlet Oxygen Generation

The absorption and emission maxima (λ_max_Ab and λ_max_Fl), extinction coefficients (ε), fluorescence quantum yields (Φ_F_), and quantum yields of the singlet oxygen generation (Φ_Δ_) of the obtained cyanines (*c*_Dye_~1 μM) measured in methanol and in aqueous saline containing 0.7% DMSO are given in Table 1, while the corresponding absorption and emission spectra are shown in Figure S1 (see Supplementary Information).

All the investigated dyes absorb and emit in the near-IR region (740–777 nm). The introduction of the iodine atom in the “parent” **Cy7COOH** results in a slight red-shift in the absorption emission maxima (up to 10 nm) in both solvents.

As compared to the previously investigated highly aggregated ***n*ICy7COOH** cyanines of a similar structure that contained two and more iodine atoms (Figure 1, *n* = 2–6) [32], the absorption spectra of the non-iodinated (**Cy7COOH**) and all the mono-iodinated **ICy7** dyes show only a negligible aggregation in saline, while both types of dyes exhibit no signs of aggregation in methanol. The most aggregative dye is **ICy7CONHPr** that contains the neutral amide group: it shows pronounced aggregation bands at both the short- and long-wavelength slopes of the main absorption band.

The extinction coefficients of all the dyes, except sulfonated **ICy7SO_3_H**, recorded in aqueous saline (ε~109,000–187,000 M^−1^cm^−1^) are approximately 1.2–2.0-fold reduced compared to those measured in methanol (ε~215,000–230,000 M^−1^cm^−1^), while the highly aggregative dye **ICy7CONHPr** exhibits the most pronounced decrease. The only exception is highly hydrophilic **ICy7SO_3_H**, which shows very similar extinction coefficient in both solvents.

The fluorescence quantum yields (Φ_F_) in aqueous saline are as much as 1.9–3.2 times lower than those in methanol. The most pronounced decrease in the quantum yield (8% in saline vs. 25.4% in methanol) is demonstrated by **ICy7CONHPr**; this effect can be also explained by substantial dye aggregation.

The quantum yields of the singlet oxygen generation (Table 1, Φ_Δ_) were measured in methanol and saline using **DPBF** [42] and **SOSG** [43], respectively, as singlet oxygen scavengers. As anticipated, the Φ_Δ_ values of mono-iodinated dyes (Φ_Δ_~1.7–2.9% in methanol and 15–65% in saline), in general, increase compared to those of non-iodinated **Cy7COOH** (Φ_Δ_~1.1% and 9.5%, respectively). The exception is only sulfonated dye **ICy7SO_3_H**, for which Φ_Δ_ in saline is about twice lower compared to **Cy7COOH** and 3.3-fold lower than that for **ICy7COOH**. To the best of our knowledge, such an unpredictable effect of sulfonic group on the quantum yield of singlet oxygen generation has never been previously reported. Remarkably, the Φ_Δ_ measured in methanol and saline change differently. Thus, Φ_Δ_ in methanol increases in the order of **Cy7COOH** < **ICy7NEt_3_** ≈ **ICy7COOH** < **ICy7SO_3_H** < **ICy7PPh_3_** ≈ **ICy7CONHPr** < **ICy7NH_2_**, while in saline, this sequence is different: **ICy7SO_3_H** < **Cy7COOH** < **ICy7COOH** < **ICy7NH_2_** < **ICy7NEt_3_** < **ICy7PPh_3_** < **ICy7CONHPr**. Additionally, the change in Φ_Δ_ in saline is more pronounced (from 4.5 to 65, in 14.4 times) than in methanol (from 1.1 to 2.9, in 2.6 times). No clear correlation between the Φ_Δ_ and the molecular structures of the dyes was revealed although **ICy7PPh_3_** and **ICy7CONHPr** exhibit more pronounced singlet oxygen generation efficacy compared to almost all other dyes in both solvent systems.

### 3.4. Dye Uptake by Bacteria

Uptake of a dye by cells along with the ability to generate singlet oxygen are important parameters that affect the dye phototoxicity. We have quantified the dye uptake for Gram-positive (*S. aureus*) and Gram-negative (*E. coli*) bacteria by using a spectrophotometric method described in Section 2.5. The dye uptake by *S. aureus* has a tendency to increase in the order of **ICy7NEt_3_** (0.57) < **Cy7COOH** (0.82) < **ICy7NH_2_** (2.65) < **ICy7SO_3_H** (3.04) ≈ **ICy7COOH** (3.09) < **ICy7CONHPr** (3.94) < **ICy7PPh_3_** (4.67), while for *E. coli*, it increases in the order of **Cy7COOH** (0.37) < **ICy7NEt_3_** (0.59) < **ICy7COOH** (1.35) < **ICy7PPh_3_** (2.20) ≈ **ICy7SO_3_H** (2.21) < **ICy7NH_2_** (2.31) < **ICy7CONHPr** (2.93) (Figure S2). Thus, the best uptake by *S. aureus* and *E. coli* was found for **ICy7PPh_3_** and **ICy7CONHPr**, respectively. The last dye also exhibits a very good uptake by *S. aureus*, while **ICy7NH_2_** is on the second place for *E. coli*. Remarkably, the magnitude of the increase is about the same (~8-fold) for both types of bacteria.

### 3.5. Toxicity and Phototoxicity of the Dyes

All experiments with bacteria were carried out in aqueous saline solution. Commercial 0.9% saline solution, used in this work, is known to be acidic, which is mostly due to the presence of CO_2_ [46]. The saline used in our experiments had a pH value of 5.15. On the other side, the carboxypentyl group in **Cy7COOH** and **ICy7COOH** as well as propanesulfonate group in **ICy7SO_3_H** are more acidic (e.g., the pK_a_ for hexanoic and ethanesulfonic acids are 4.88 [47] and 1.68 [48], respectively), thus indicating that the carboxylic and the sulfonic groups are mostly deprotonated and the dyes **Cy7COOH**, **ICy7COOH**, and **ICy7SO_3_H** exist in saline buffer in the non-charged zwitterion form. On the contrary, **ICy7NEt_3_** and **ICy7PPh_3_** have a charge of +1 that is localized on the triethylammonium and triphenylphosphonium groups, which results in the overall dye charge of +2. The **ICy7CONHPr** dye has a delocalized charge of +1, while the amino group in **ICy7NH_2_** is protonated providing the overall charge of +2.

To investigate the effect of the **ICy7** dyes on bacteria, the stock solutions of the dyes were prepared in DMSO and added to the bacterial suspension in saline in such a way that the concentration of DMSO in each sample was 0.7%. Then, the bacterial suspensions were incubated with each dye for 30 min in the dark (pre-irradiation incubation), exposed to light and grown in the dark for 24 h, at 37 °C, followed by the calculation of the number of bacterial colonies. The dye concentrations and the exposure time were varied for each dye.

To verify the *dark* cytotoxicity of the dyes, the same experiments were carried out in parallel without light exposure. We found that the investigated dyes have no detectable dark toxicity to *S. aureus* (Figure S3), *E. coli* (Figures S4 and S5) and *P. aeruginosa* (Figures S6 and S7) at least up to the dye concentrations of 0.5 µM for *S. aureus* and 50 µM for *E. coli* and *P. aeruginosa*.

In the next step, the effect of three main parameters on the *photodynamic* eradication of bacteria was investigated: (i) the structure of the solubilizing group in the dye molecules, (ii) the dye concentration, and (iii) the light exposure doses (the light power multiplied by the irradiation time).

#### 3.5.1. Photodynamic Eradication of *S. aureus*

The effect of the solubilizing groups in the **ICy7** dyes was first investigated on Gram-positive bacteria *S. aureus* at constant light dose (3 J/cm^2^) vs. the dye concentrations (*c*_Dye_ = 0.01–0.5 µM). The obtained results were compared to those for the reference non-iodinated **Cy7COOH**. As expected, iodinated dyes **ICy7COOH**, **ICy7CONHPr**, **ICy7PPh_3_**, and **ICy7NH_2_** demonstrate an elevated phototoxicity as compared to the non-iodinated **Cy7COOH** (the exceptions are **ICy7SO_3_H** and **ICy7NEt_3_**, which are discussed below). Thus, **ICy7COOH** at *c*_Dye_ = 0.5 μM eradicates *S. aureus* 2.9-fold more efficiently (26.8% survival) than **Cy7COOH** (76.6% survival).

**Figure 3:**
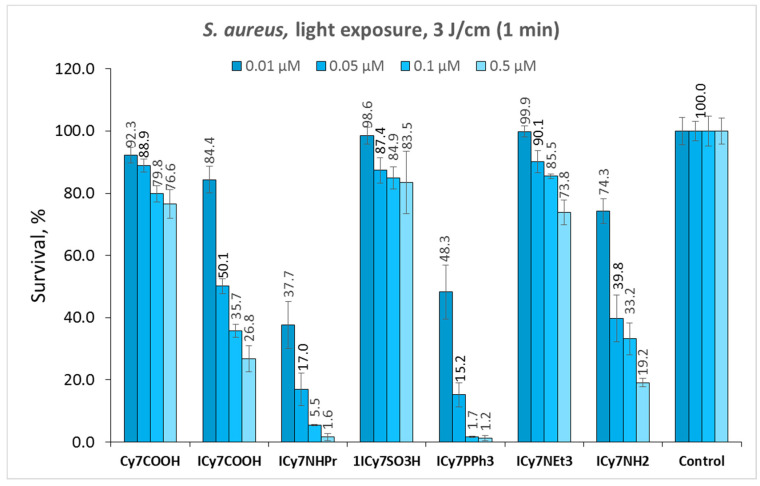
Survival of *S. aureus* in saline containing 0.7% DMSO, when exposed to 3 J/cm^2^ light dose (56 mW/cm^2^, 1 min) vs. the dye concentrations. Light-irradiated cells without dyes were used as a control.

The replacement of the negatively charged carboxylic group in **ICy7COOH** with the neutral N-propylamide group in **ICy7CONHPr** and positively charged triphenylphosphonium group in **ICy7PPh_3_** leads to the noticeable increase in phototoxicity with only 1.6% and 1.2% survival, respectively (Figure 3, *c*_Dye_ = 0.5 μM; light dose 3 J/cm^2^). This result can be explained by the most pronounced Φ_Δ_ (Table 1) and preferential uptake of these positively charged dyes by *S. aureus* (Section 3.4).

Surprisingly, the **ICy7NH_2_** and **ICy7NEt_3_** with an overall charge of +2 show noticeably reduced phototoxicity compared to **ICy7PPh_3_** bearing the same charge. Moreover, **ICy7NH_2_** produces about the same phototoxic effect as the neutral **ICy7COOH**, while the **ICy7NEt_3_** has about the same phototoxicity as non-iodinated **Cy7COOH**. Unexpectedly, the zwitterionic dye **ICy7SO_3_H** exhibiting excellent penetration in *S. aureus* (Section 3.4) shows the least pronounced phototoxicity with a survival percentage of more than 80% at all the studied dye concentrations. This can be explained by the extremely low Φ_Δ_ for this dye (4.5% in saline, Table 1).

The increase in the dye concentration results in elevating phototoxicity of all the investigated dyes although for the lower-effective **Cy7COOH**, **ICy7COOH**, **ICy7SO_3_H**, and **ICy7NH_2_** this increase is less pronounced.

**Figure 4:**
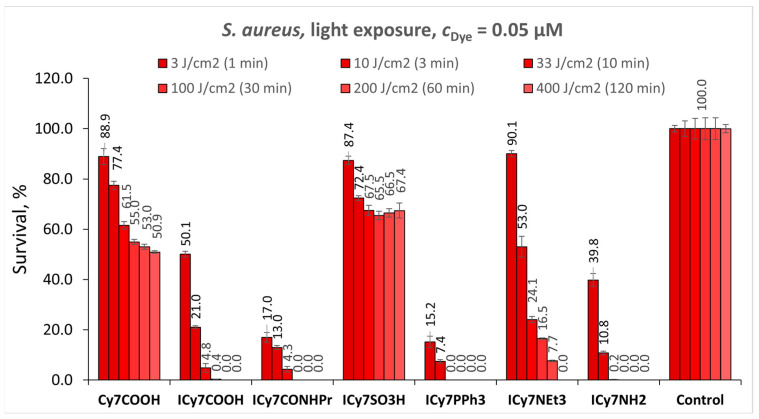
Survival of *S. aureus* at (*c*_Dye_ = 0.05 µM in saline containing 0.7% DMSO), when exposed to 3 J/cm^2^ (1 min), 10 J/cm^2^ (3 min), 33 J/cm^2^ (10 min), 100 J/cm^2^ (30 min), 200 J/cm^2^ (60 min), and 400 J/cm^2^ (120 min) light doses.

Furthermore, we studied the effect of the light dose between 3 J/cm^2^ and 400 J/cm^2^ on the phototoxicity of the dyes towards *S. aureus* at constant dye concentration of 0.05 µM (Figure 4). We found that the phototoxicity of **ICy7COOH**, **ICy7CONHPr**, **ICy7NEt_3_**, **ICy7PPh_3_**, and **ICy7NH_2_**, substantially increases when raising the light dose, but this increase is much less pronounced for the non-iodinated **Cy7COOH** and sulfonated **ICy7SO_3_H**. Noteworthy, **ICy7COOH** and **ICy7CONHPr** almost totally eradicate *S. aureus* at 100 J/cm^2^ (30 min irradiation), while **ICy7PPh_3_** and **ICy7NH_2_** are even more effective; they completely kill this pathogen at 33 J/cm^2^ (10 min). As shown in Figure 4, the iodinated dyes bearing a positive +1 (**ICy7CONHPr**) or +2 (**ICy7PPh_3_** and **ICy7NH_2_**) charges cause the more pronounced eradication of these bacteria, while zwitterionic dye **ICy7SO_3_H** bearing negatively charged sulfonic group is much less phototoxic. Remarkably, both **ICy7PPh_3_** and **ICy7NEt_3_** bear a positive charge, but the first one is much more phototoxic compared to the second one.

To conclude, **ICy7PPh_3_** is the most effective photosensitizer against *S. aureus* at a low concentration of 0.05 µM and low light dose of 33 J/cm^2^ (10 min). **ICy7COOH**, **Cy7CONHPr**, **ICy7NEt_3_**, and **ICy7NH_2_** require either a larger concentration or higher light dose for complete bacteria eradication.

#### 3.5.2. Photodynamic Eradication of *E. coli* and *P. aeruginosa*

**Figure 5:**
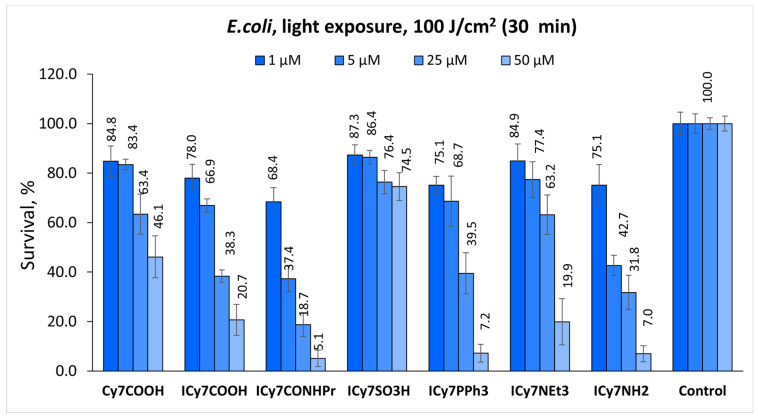
Survival of *E. coli* in saline containing 0.7% DMSO, when exposed to 100 J/cm^2^ light dose (56 mW/cm^2^, 30 min) vs. the dye concentrations.

**Figure 6:**
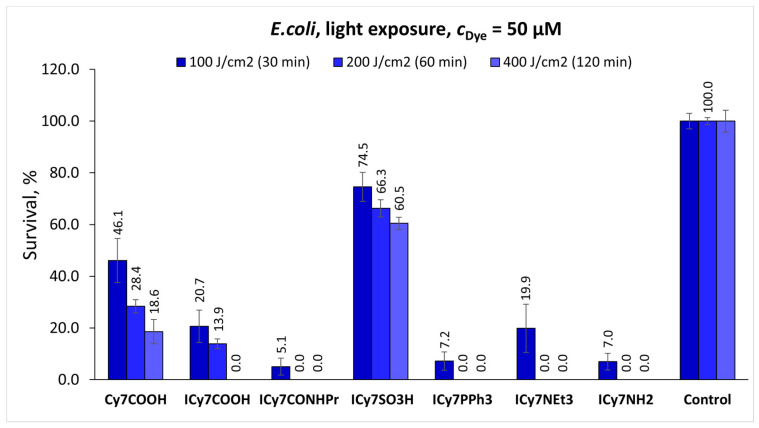
Survival of *E. coli* at (*c*_Dye_ = 50 µM in saline containing 0.7% DMSO), when exposed to 100 J/cm^2^ (30 min), 200 J/cm^2^ (60 min), and 400 J/cm^2^ (120 min) light doses.

**Figure 7:**
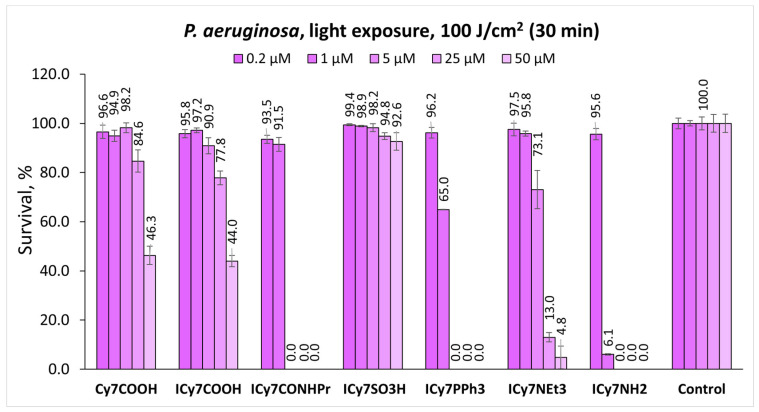
Survival of *P. aeruginosa* in saline containing 0.7% DMSO, when exposed to 100 J/cm^2^ light dose (56 mW/cm^2^, 30 min) vs. the dye concentrations.

**Figure 8:**
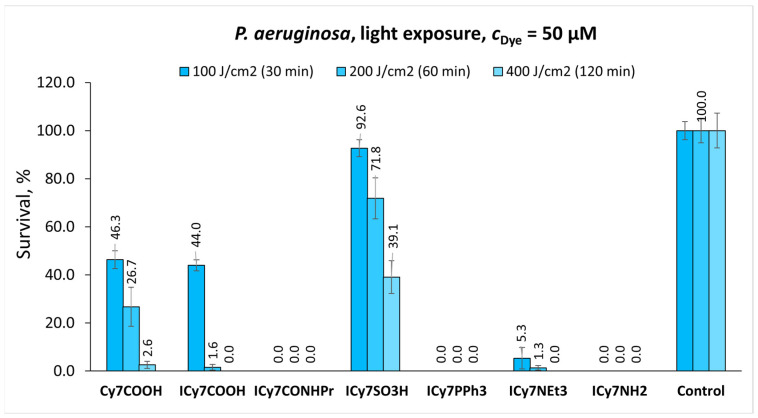
Survival of *P. aeruginosa* at (*c*_Dye_ = 50 µM in saline containing 0.7% DMSO), when exposed to 100 J/cm^2^ (30 min), 200 J/cm^2^ (60 min), and 400 J/cm^2^ (120 min) light doses.

In the next step, we studied phototoxicity of the dyes towards Gram-negative pathogens *E. coli* and *P. aeruginosa* (Figure 5, Figure 6, Figure 7 and Figure 8). We investigated the effect of the dye concentration at the constant light dose of 100 J/cm^2^ (Figure 5 and Figure 7) and the impact of the light dose at the constant dye concentration of 50 µM (Figure 6 and Figure 8) for both *E. coli* (Figure 5 and Figure 6) and *P. aeruginosa* (Figure 7 and Figure 8). These bacteria were found to be much more resistant towards photodynamic treatment compared to Gram-positive *S. aureus* (Figure 3 and Figure 4). Thus, a reasonable phototoxicity was observed for the dye concentration of 5–50 µM, which was two–three orders higher compared to that used for *S. aureus* (0.05 µM) and at the higher light dose of 100 J/cm^2^.

Similar to Gram-positive *S. aureus*, both types of Gram-negative bacteria are most resistant towards **ICy7SO_3_H** exhibiting the survival of 60.5% on *E. coli* and 39.1% on *P. aeruginosa* (light dose 400 J/cm^2^). Remarkably, this dye was found to be much less phototoxic than the non-iodinated **Cy7COOH** (survival 18.6% for *E. coli* and 2.6% for *P. aeruginosa* at 400 J/cm^2^). The most effective photokillers are **ICy7CONHPr**, **ICy7PPh_3_** and **ICy7NH_2_**, while **ICy7NEt_3_** and **ICy7COOH** are a bit less phototoxic. All these dyes are more active towards *E. coli* and *P. aeruginosa* at 100 J/cm^2^ (Figure 5 and Figure 7) compared to **ICy7SO_3_H** and **Cy7COOH**; and the difference in the phototoxicity between them is even more pronounced at 200–400 J/cm^2^ (Figure 6 and Figure 8). The investigated pathogens can be totally eradicated by **ICy7CONHPr**, **ICy7PPh_3_**, and **ICy7NH_2_** at the dye concentration of 50 µM and light dose of 200 J/cm^2^ for *E. coli*, while 5 µM and 100 J/cm^2^ are sufficient for the complete eradication of *P. aeruginosa*.

The introduction of the positively charged triphenylphosphonium (**ICy7PPh_3_**) and triethylammonium (**ICy7NEt_3_**) groups causes a noticeable increase in the phototoxic effect on both Gram-negative pathogens, especially at higher light doses of 200–400 J/cm^2^. Interestingly, while **ICy7NEt_3_** exhibited a poor phototoxicity on *S. aureus* (Figure 3), it performs very well on *E. coli* and *P. aeruginosa* (Figure 5, Figure 6, Figure 7 and Figure 8).

To summarize, the positively charged dyes are more effective against *E. coli* and *P. aeruginosa*, and their efficacy increases in the order of **ICy7NEt_3_** < **ICy7NH_2_** ≈ **ICy7PPh_3_** ≈ **ICy7CONHPr**.

#### 3.5.3. Phototoxicity of the Dyes vs. the Quantum Yield of the Singlet Oxygen Generation and the Uptake by Bacteria

**Figure 9:**
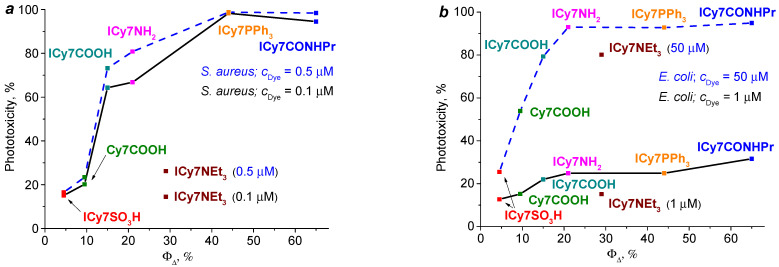
Phototoxicity of the dyes on *S. aureus* (**a**) and *E. coli* (**b**) at 3 J/cm^2^ and 100 J/cm^2^ light doses, respectively, vs. the quantum yield of the singlet oxygen generation (Φ_Δ_) in saline.

We correlated the phototoxicity of the dyes (Phototoxicity = 100% − Cell Survival) with their quantum yields of the singlet oxygen generation at two different dye concentrations (Figure 9). It was found that the Φ_Δ_ and phototoxicity on *S. aureus* (Figure 9a) and *E. coli* (Figure 9b) simultaneously increase in the order: **ICy7SO_3_H** < **Cy7COOH** < **ICy7COOH** < **ICy7NH_2_** ≤ **ICy7PPh_3_** ≈ **ICy7CONHPr**. Remarkably, **ICy7CONHPr** and **ICy7PPh_3_** demonstrate the best Φ_Δ_ and the most pronounced phototoxicity on both types of bacteria, while **ICy7NEt_3_** shows a reduced phototoxicity compared to the anticipated value based on its Φ_Δ_ (there is a deviation from the average curve in Figure 9).

**Figure 10:**
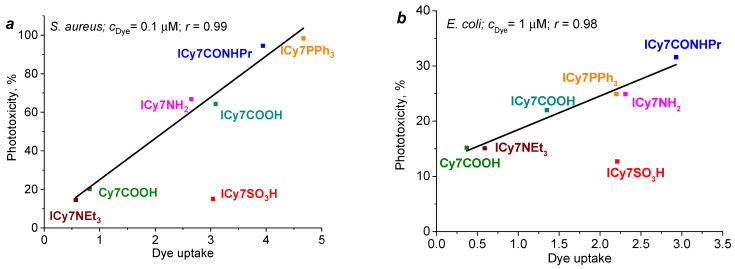
Phototoxicity of the dyes vs. the uptake by *S. aureus* at *c*_Dye_ = 0.1 µM (**a**) and *E. coli* at *c*_Dye_ = 1 µM (**b**). The light doses are 3 J/cm^2^ and 100 J/cm^2^, respectively.

Furthermore, we correlated the phototoxicity of the dyes with their uptake by *S. aureus* and *E. coli* (Figure 10). It can be seen that there is an excellent linear correlation between the phototoxicity and the uptake (*r* = 0.99 for *S. aureus* and *r* = 0.98 for *E. coli*) for all the investigated dyes except the sulfonated **ICy7SO_3_H** that exhibits a good uptake with a notably reduced phototoxicity.

Taking into account the obtained data on the dye uptake (Figure 10) and Φ_Δ_ (Table 1 and Figure 9), it can be concluded that the reduced phototoxicity of **Cy7SO_3_H** is connected with its insufficient Φ_Δ_, while the low phototoxicity of **ICy7NEt_3_** and **Cy7COOH** is due to the decreased uptake.

## 4. Conclusions

In summary, a series of novel iodinated heptamethine cyanine dyes containing various solubilizing groups attached to one of the quaternized indolenine moieties was synthesized. Their spectral properties, the yields of singlet oxygen generation, uptake by bacteria, dark cytotoxicity and phototoxicity against selected Gram-positive and Gram-negative pathogenic bacteria were investigated. The structure of the solubilizing group was found to have a strong effect on the dye uptake and phototoxicity. All the dyes exhibit a negligible cytotoxicity in the dark at the concentrations of at least up to 0.5 µM for *S. aureus* and 50 µM for *E. coli* and *P. aeruginosa*. The dyes containing neutral and positively charged groups demonstrate a high phototoxicity at nanomolar and micromolar dye concentrations against Gram-positive and Gram-negative bacteria, respectively, while the dyes with negatively charged groups have a reduced phototoxicity. The most efficient photosensitizers are positively charged iodinated heptamethine cyanines **ICy7CONHPr** and **ICy7PPh_3_**. These dyes completely kill *S. aureus* at 0.05 µM and low NIR light dose of 33 J/cm^2^, *E. coli* at 50 µM and 200 J/cm^2^, and *P. aeruginosa* at 5 µM and 100 J/cm^2^.

Thus, a combination of the heavy-atom effect that increased singlet oxygen generation with an appropriate solubilizing group’s effect improving cell uptake helped to noticeably increase the APDT efficacy of these new photosensitizers.

We believe that the obtained dyes **ICy7CONHPr** and **ICy7PPh_3_** can be further exploited for the development of highly efficient sensitizing systems for photo-eradication of bacteria, viruses, and abnormal cells; equipping these photosensitizers with bacteria-specific carriers such as bacterial substrates or antibodies can pave the way for their clinical use.

## Figures and Tables

**Table 1:** Spectral characteristics of the obtained cyanine dyes (*c*_Dye_~1 µM) measured in methanol and in aqueous saline containing 0.7% DMSO.

Dye Structure	MeOH					0.7% DMSO in Saline				
	λ_max_Ab, nm	ε M^−1^cm^−1^	λ_max_Fl, nm	Φ_F_^1^, %	Φ_Δ_^2^, %	λ_max_Ab, nm	ε, M^−1^cm^−1^	λ_max_Fl, nm	Φ_F_^1^, %	Φ_Δ_^3^, %
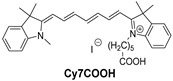	744	230,000	774	25.3 ± 0.5	1.1 ± 0.1	740	169,000	768	13 ± 1	9.5 ± 0.6
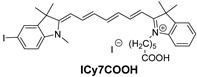	749	230,000	780	25.8 ± 0.5	1.8 ± 0.2	747	143,000	776	11 ± 2	15 ± 1
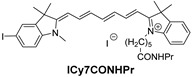	749	215,000	784	25.4 ± 0.4	2.5 ± 0.1	747	109,000	776	8 ± 1	65 ± 4
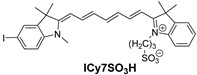	750	230,000	781	25.5 ± 0.8	2.0 ± 0.1	747	238,000	777	10 ± 1	4.5 ± 0.3
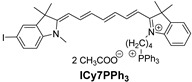	751	230,000	781	25.9 ± 0.5	2.5 ± 0.2	750	155,000	777	9.1 ± 0.8	44 ± 3
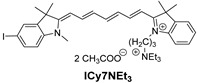	747	230,000	778	24.7 ± 0.7	1.7 ± 0.2	745	176,000	775	12 ± 1	29 ± 1
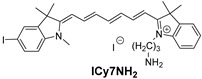	749	220,000	780	24.6 ± 0.6	2.9 ± 0.1	745	187,000	775	9 ± 1	21 ± 2

^1^ **Cy7**^TM^ was used as the reference, Φ_F_ = 13% in PBS, pH = 7.4. ^2^ **HITC** was used as the reference, Φ_Δ_ = 0.89% in methanol. ^3^ **ICG** was used as the reference, Φ_Δ_ = 0.2% in PBS, pH = 7.4.

## Data Availability

Data is contained within this article and Supplementary Materials.

## Supplementary Materials

The following supporting information can be downloaded at: https://www.mdpi.com/article/10.3390/pharmaceutics15010247/s1.
