# Supplementary material for: Effect of Solubilizing Group on the Antibacterial Activity of Heptamethine Cyanine Photosensitizers
Source: Pharmaceutics. 2023 Jan 11;15(1):247. doi: 10.3390/pharmaceutics15010247 (PMC9864305; doi:10.3390/pharmaceutics15010247)
Supplement: Supplementary file 1 [file pharmaceutics-15-00247-s001.zip › pharmaceutics-2048149-supplementary.pdf]

Supplementary information

Effect of Solubilizing Group on the Antibacterial Activity of Heptamethine Cyanine Photosensitizers

Olga Semenova[a], Dmytro Kobzev[a], Iryna Hovor[a,b], Melad Atrash[b], Faina Nakonechny[b], Olesia Kulyk[a], Andrii Bazylevich[a], Gary Gellerman[a], Leonid Patsenker[a,*]

[a] *Department of Chemical Sciences, Ariel University, Ariel 40700,, Israel*

[b] *Department of Chemical Engineering, Ariel University, Ariel 40700,, Israel*

*Corresponding author:

Leonid Patsenker,

Department of Chemical Sciences,

Ariel University, Ariel 40700, Israel

E-mail address: leonidpa@ariel.ac.il

Table of contents

1. UV-Vis and fluorescence spectra........S1
2. Data on dye uptake and photodynamic eradication in the dark........S3
3. ^{1}H NMR spectra........S6
4. ^{13}C NMR spectra........S13
5. HRMS spectra........S20
6. HPLC data........S27

1. UV-Vis and fluorescence spectra

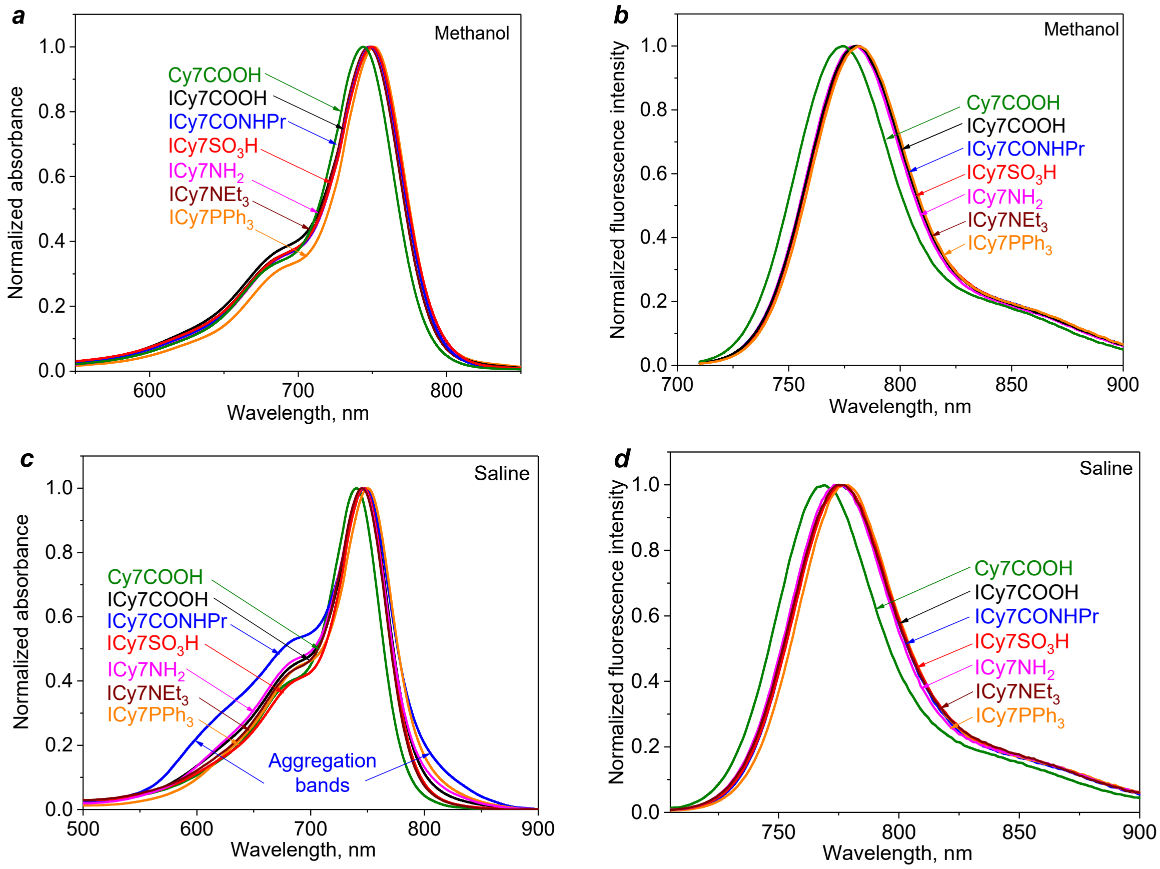

Figure S1. Absorption (***a***, ***c***) and emission (***b***, ***d***) spectra of cyanine dyes ($c \sim 1$ µM) in methanol (***a***, ***b***, λ* 680 nm) and aqueous saline (***c***, ***d***, λ* 700 nm).

2. Data on dye uptake and photodynamic eradication in the dark

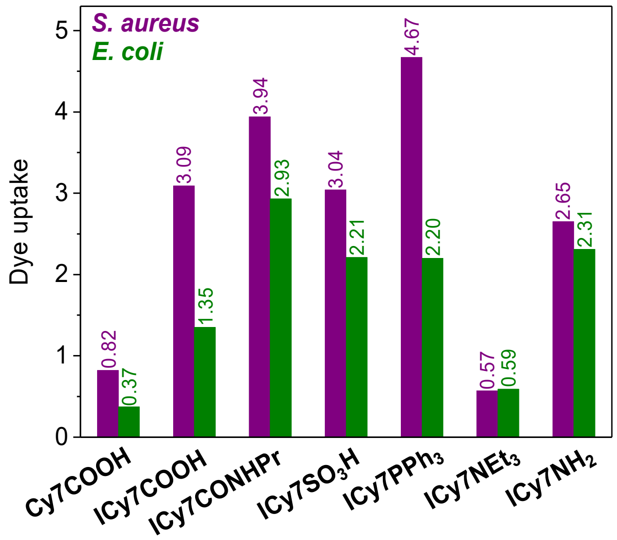

Figure S2. The uptake of dyes (c_{Dye} = 1 µM) by *S. aureus* and *E. coli*.

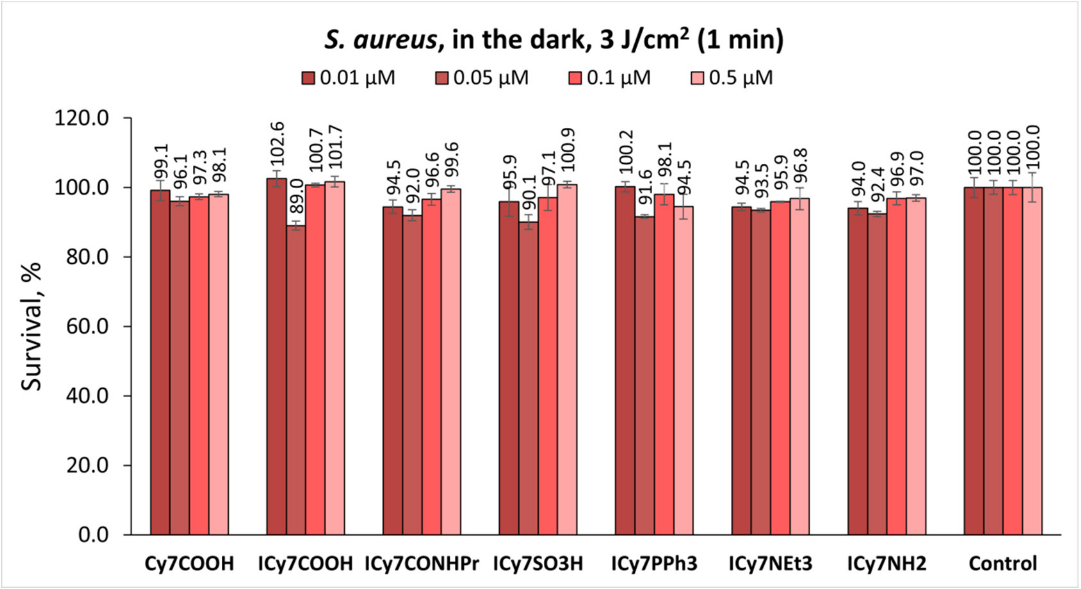

Figure S3. Survival of *S. aureus* in 0.7% DMSO in saline in the dark *vs.* the dye concentrations.

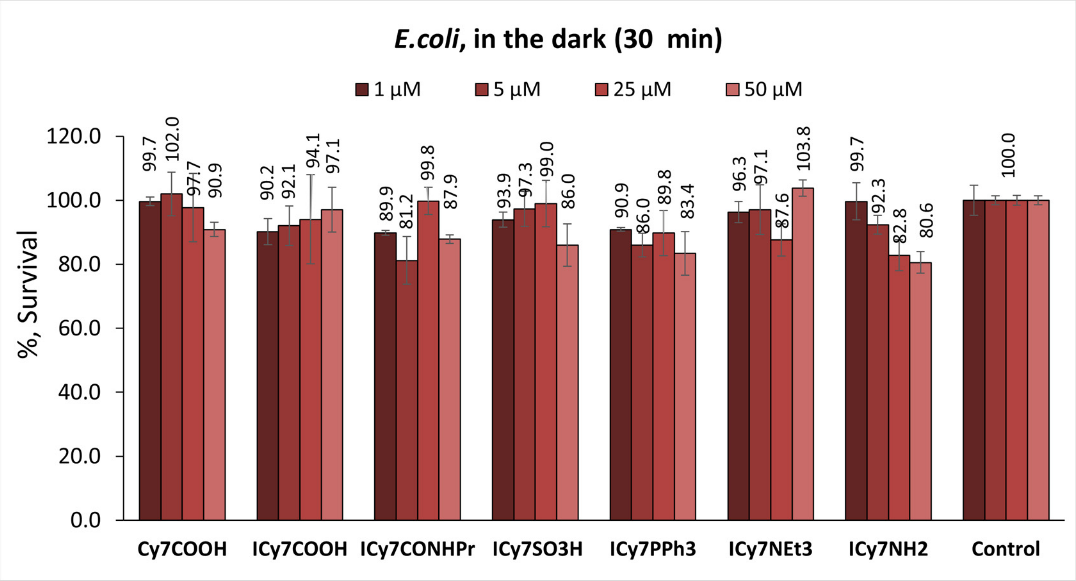

Figure S4. Survival of *E. coli* in 0.7% DMSO in saline in the dark *vs.* the dye concentrations.

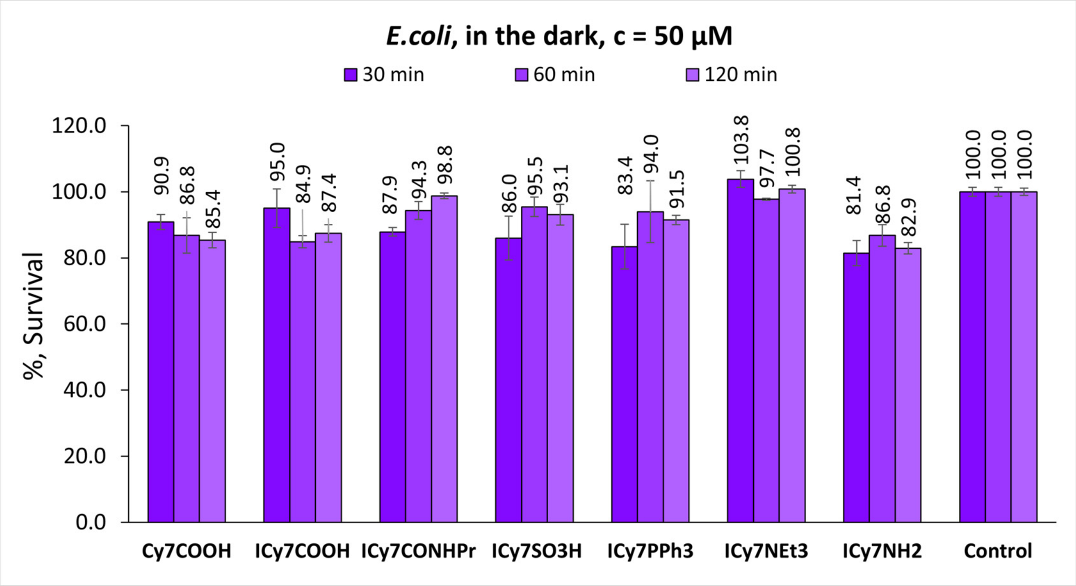

Figure S5. Survival of *E. coli* at 50 µM dye concentrations in 0.7% DMSO in saline for 30, 60 and 120 min in the dark.

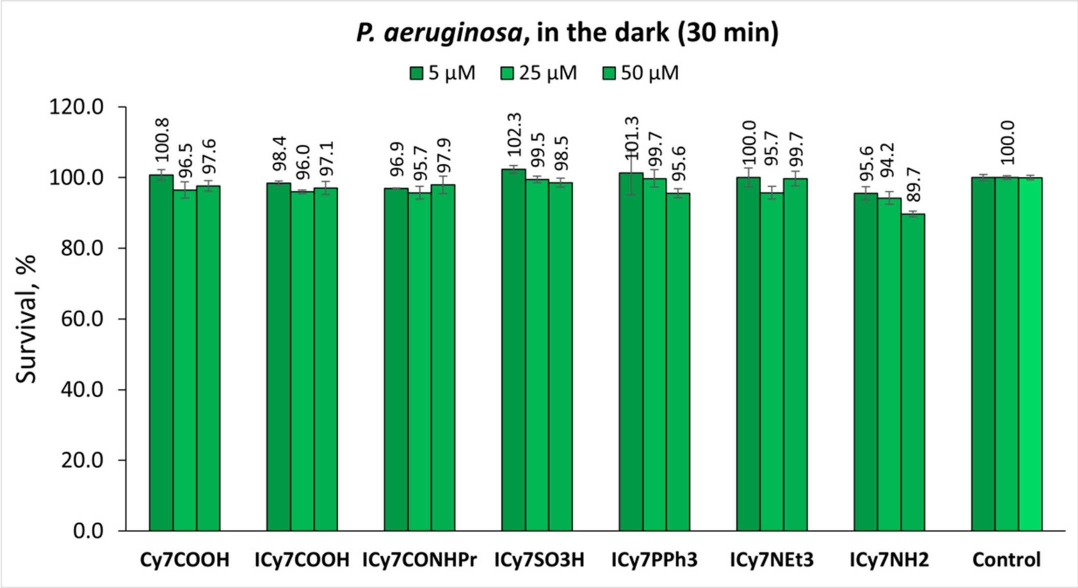

Figure S6. Survival of *P. aeruginosa* in 0.7% DMSO in saline in the dark *vs.* the dye concentrations.

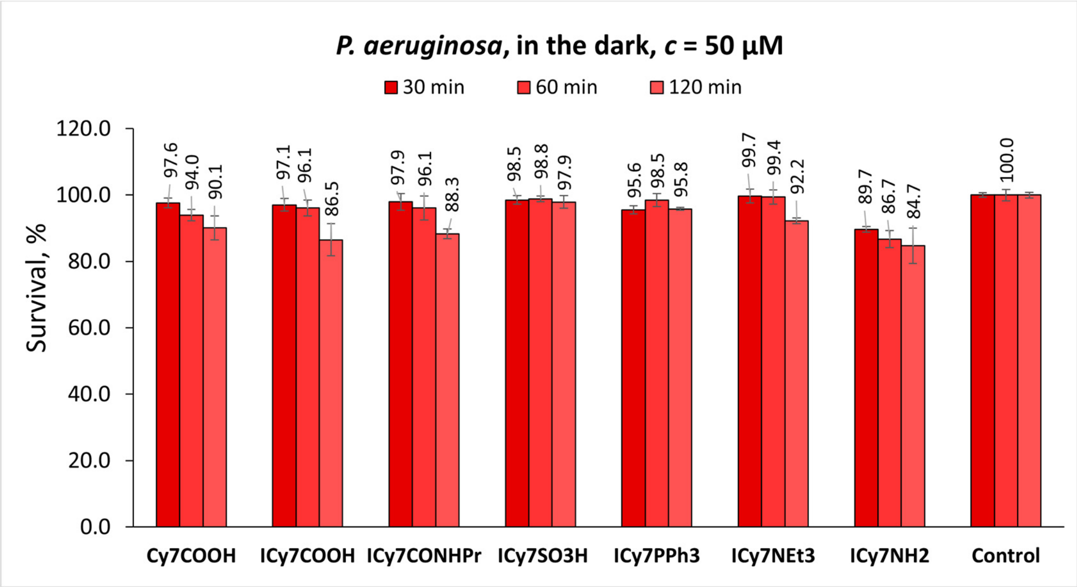

Figure S7. Survival of *P. aeruginosa* at 50 µM dye concentrations in 0.7% DMSO in saline for 30, 60 and 120 min in the dark.

3. ^{1}H NMR spectra

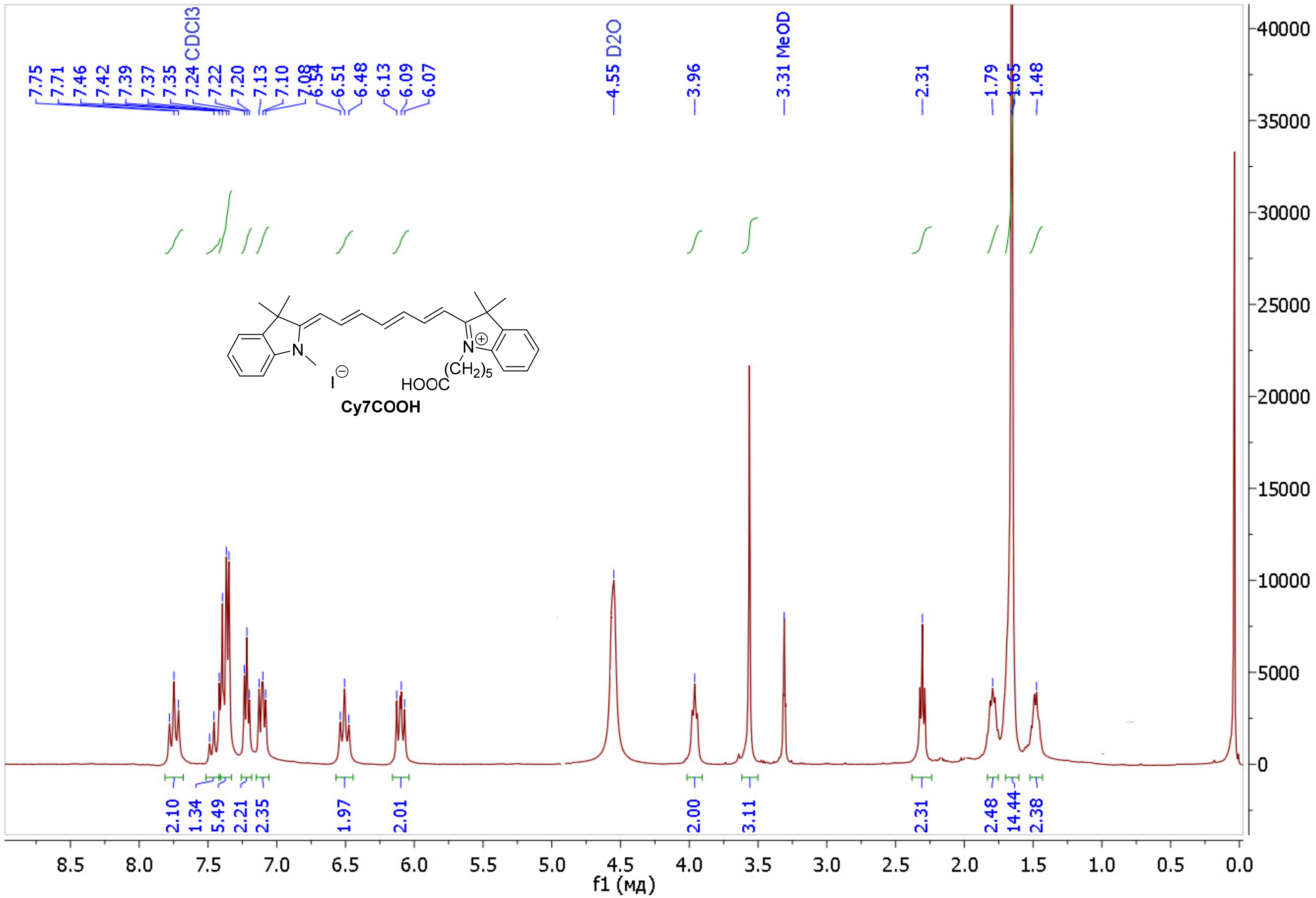

Figure S8. ^{1}H NMR spectrum of **Cy7COOH** (400 MHz, $CDCl_3$–CD_3OD).

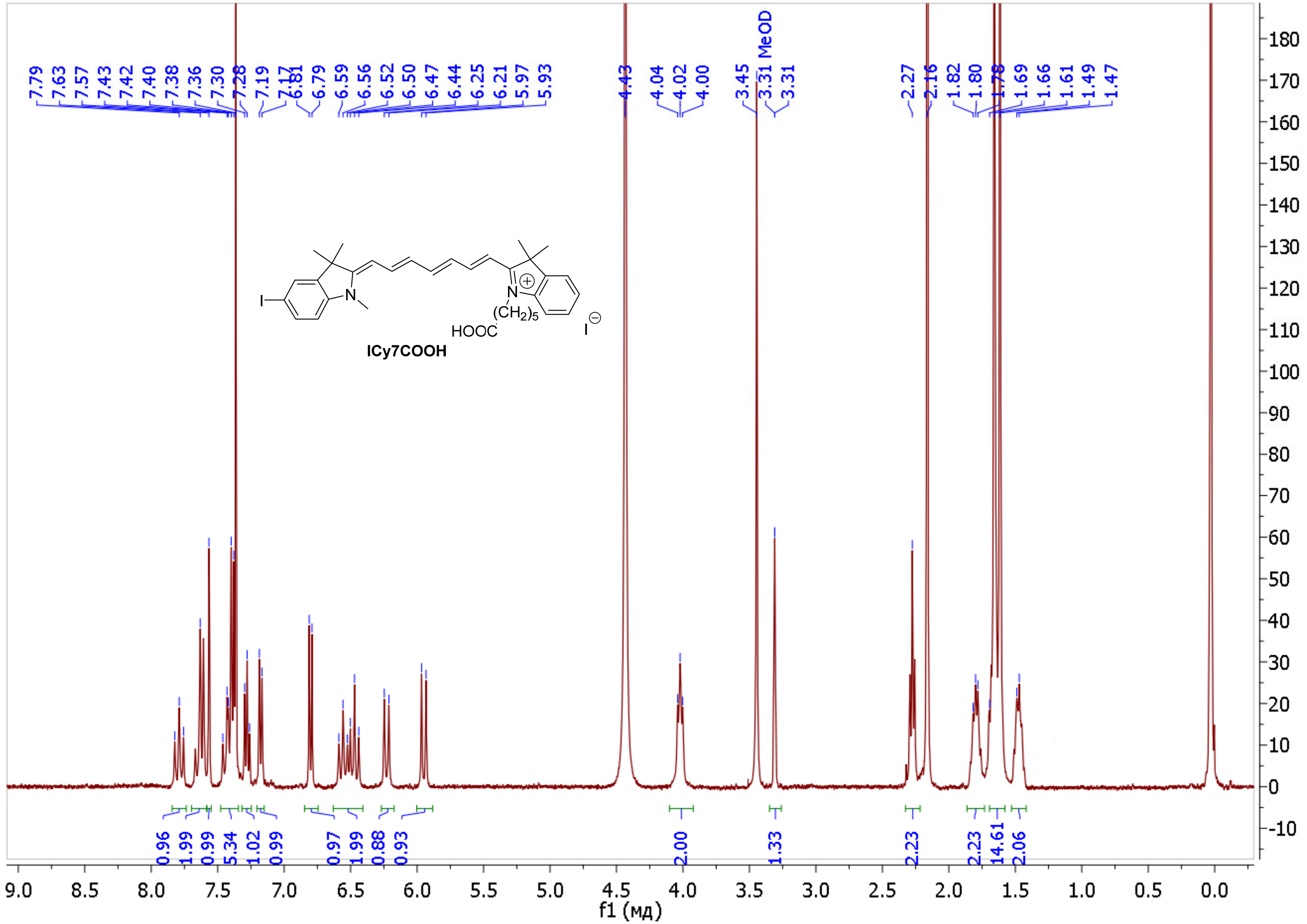

Figure S9. ^{1}H NMR spectrum of **ICy7COOH** (400 MHz, $CDCl_3$–CD_3OD).

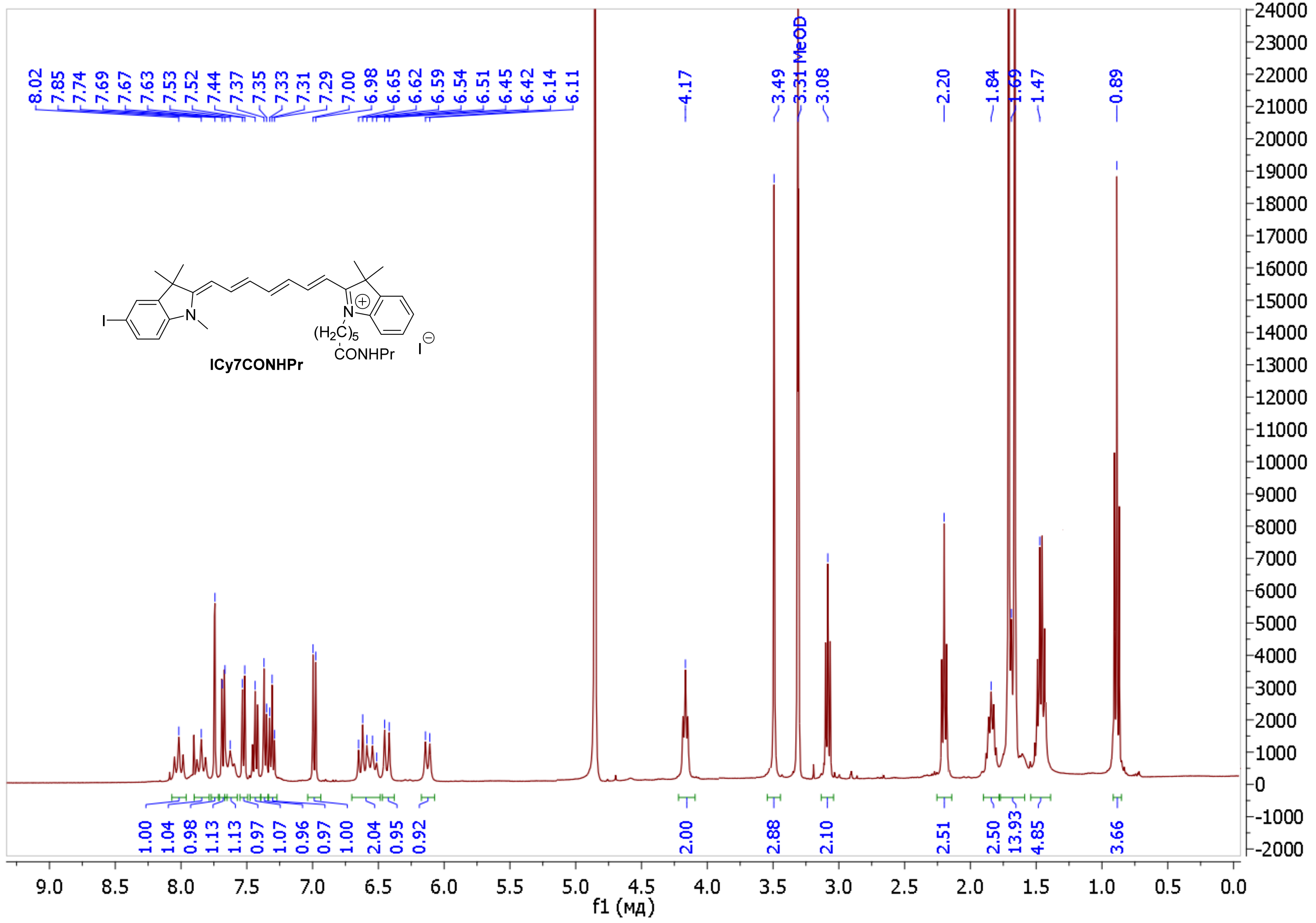

Figure S10. ^{1}H NMR spectrum of **ICy7CONHPr** (400 MHz, CD_3OD).

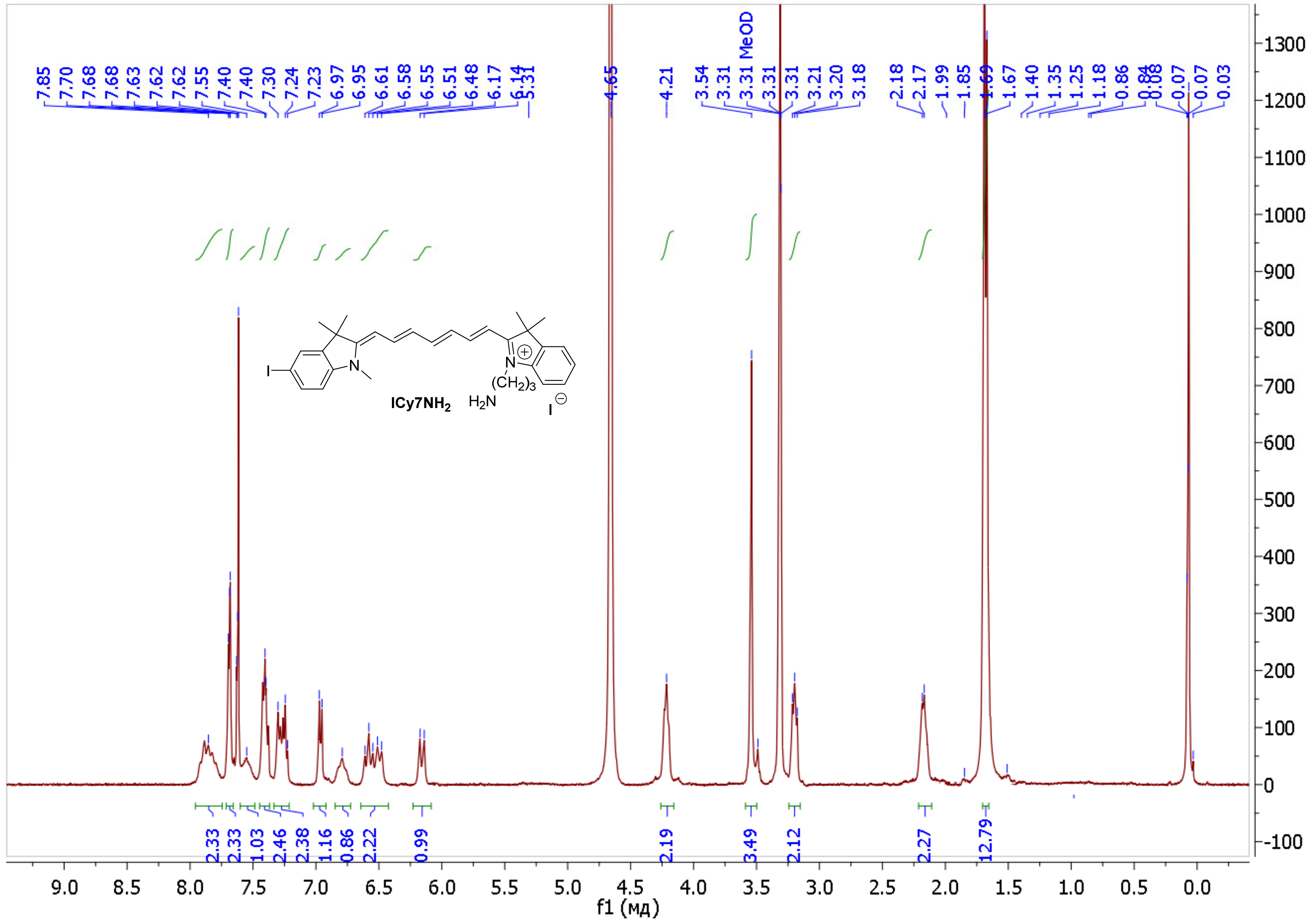

Figure S11. ^{1}H NMR spectrum of **ICy7NH₂** (400 MHz, $CDCl_3$–CD_3OD).

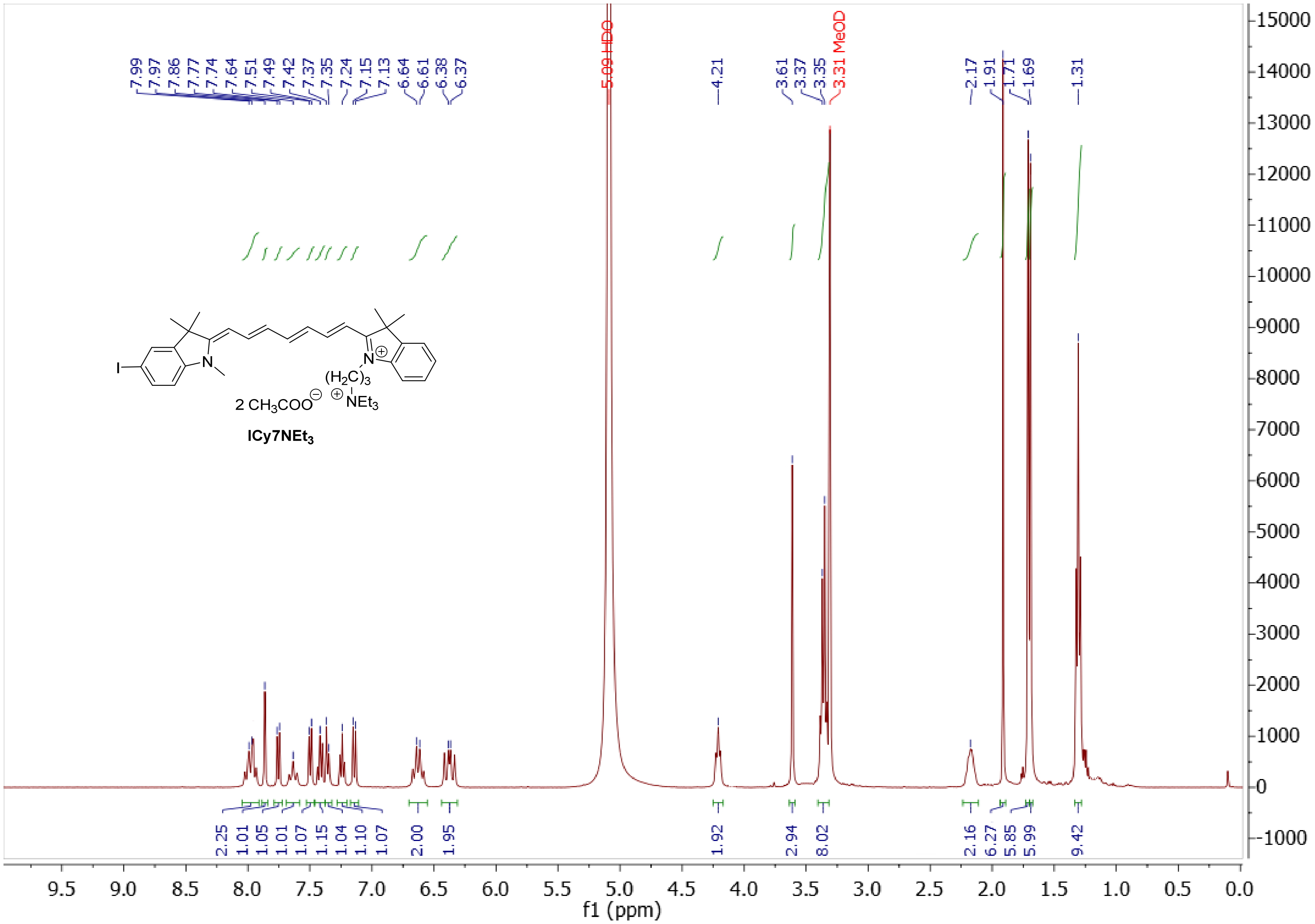

Figure S12. ^{1}H NMR spectrum of **ICy7NEt$_3$** (400 MHz, CD_3OD).

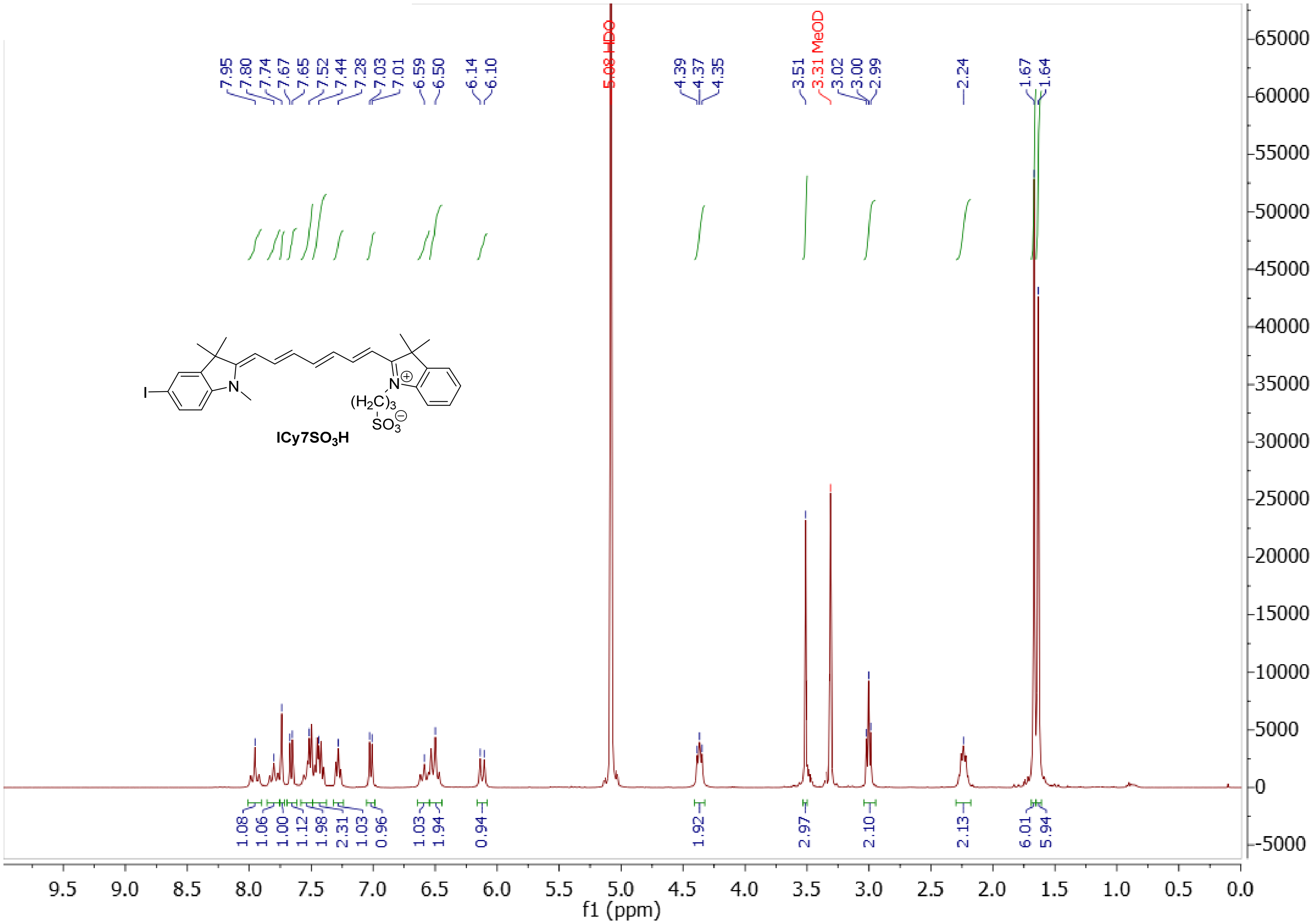

Figure S13. ^{1}H NMR spectrum of **ICy7SO$_3$H** (400 MHz, CD_3OD).

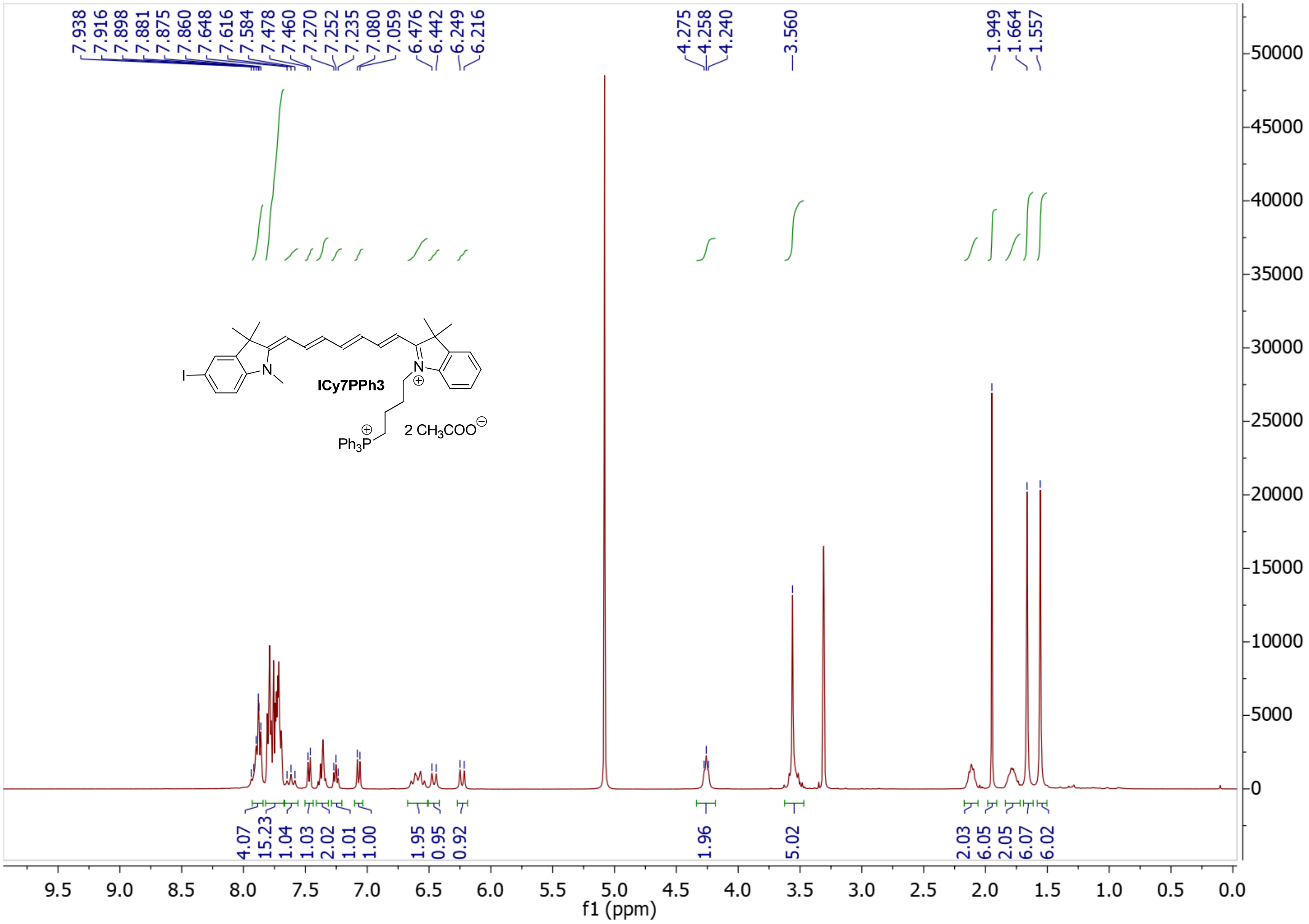

Figure S14. ^{1}H NMR spectrum of **ICy7PPh$_3$** (400 MHz, CD_3OD).

4. ^{13}C NMR spectra

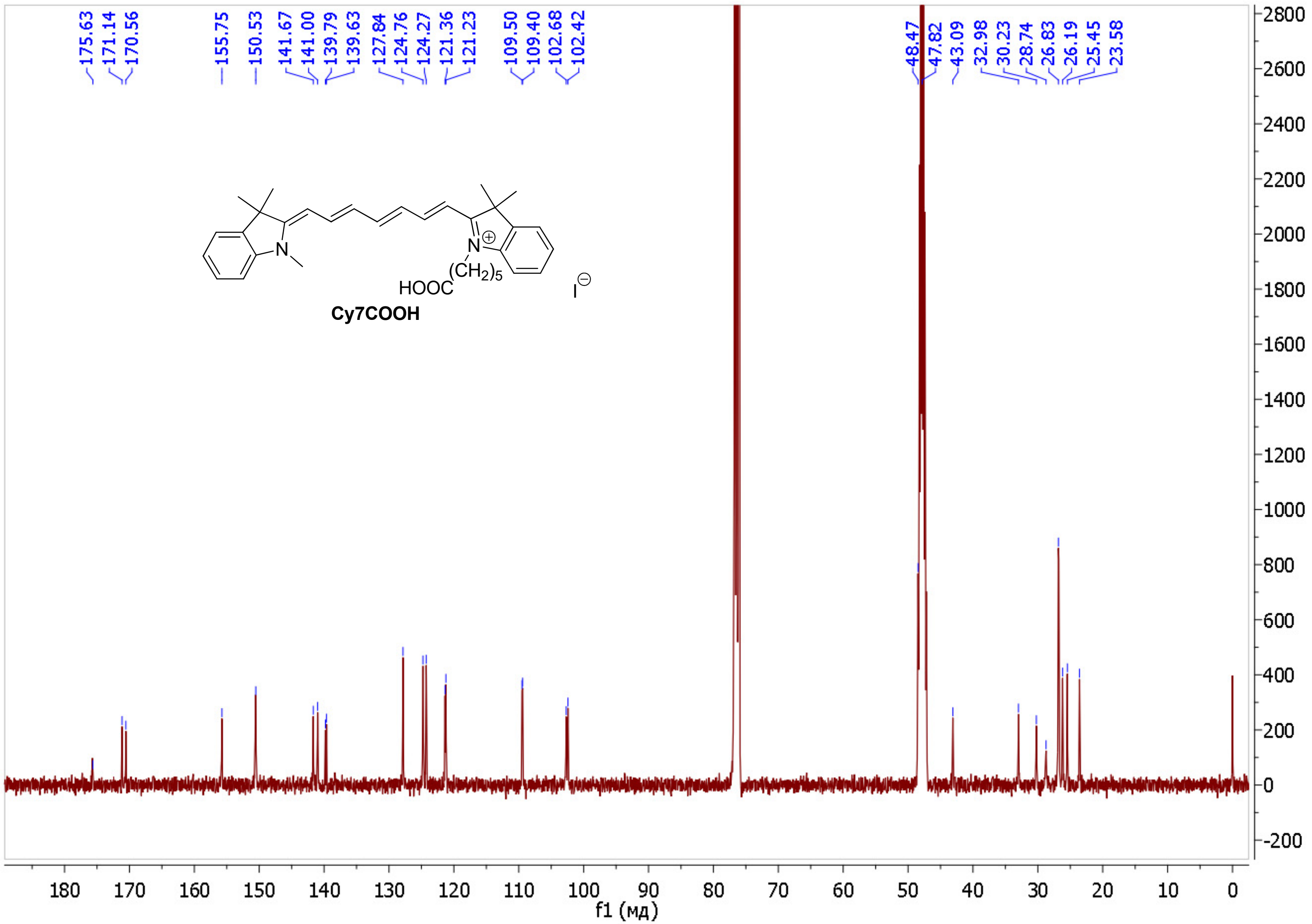

Figure S15. ^{13}C NMR spectrum of **Cy7COOH** (100 MHz, $CDCl_3$–CD_3OD).

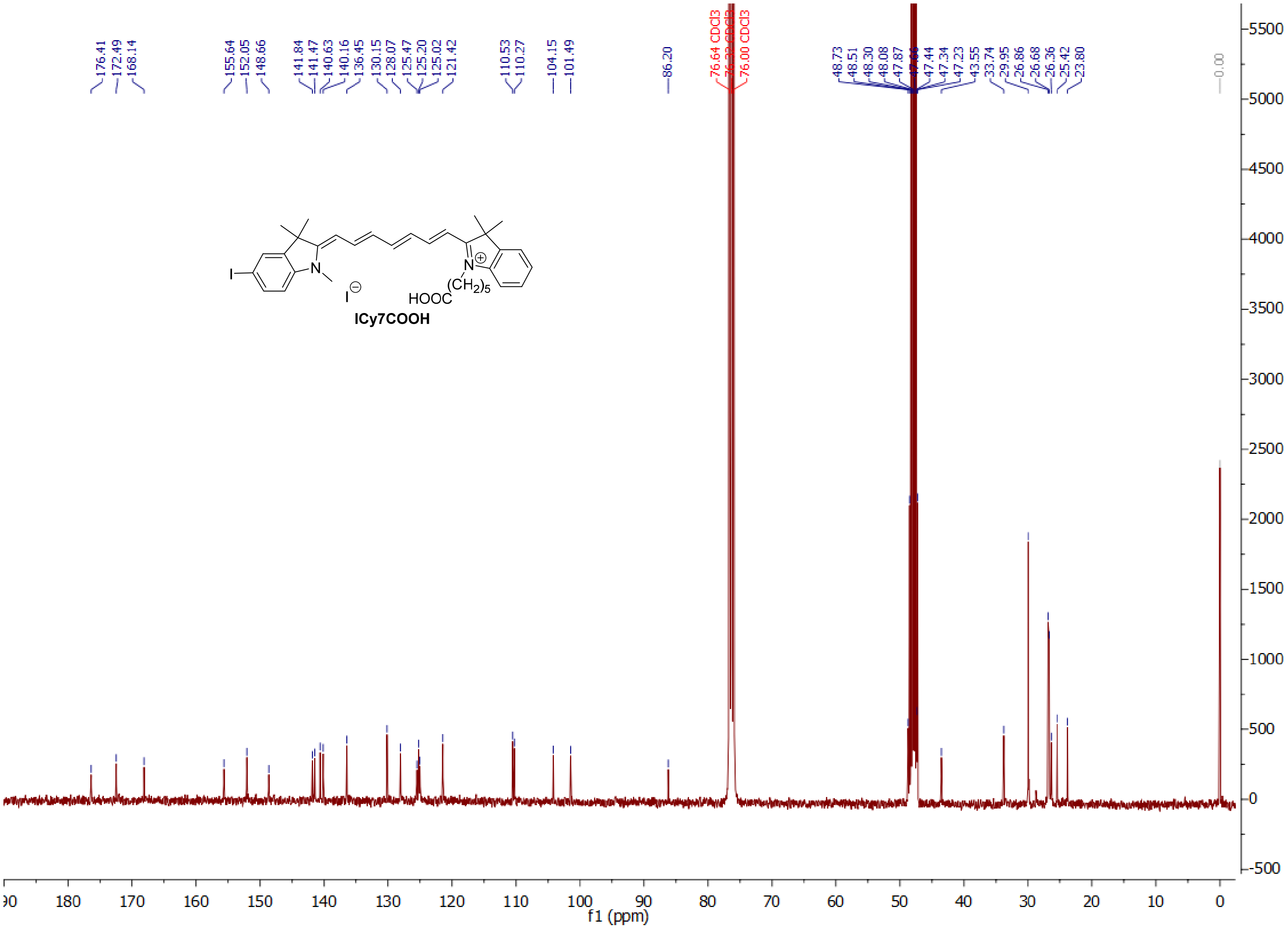

Figure S16. ^{13}C NMR spectrum of **ICy7COOH** (100 MHz, $CDCl_3$–CD_3OD).

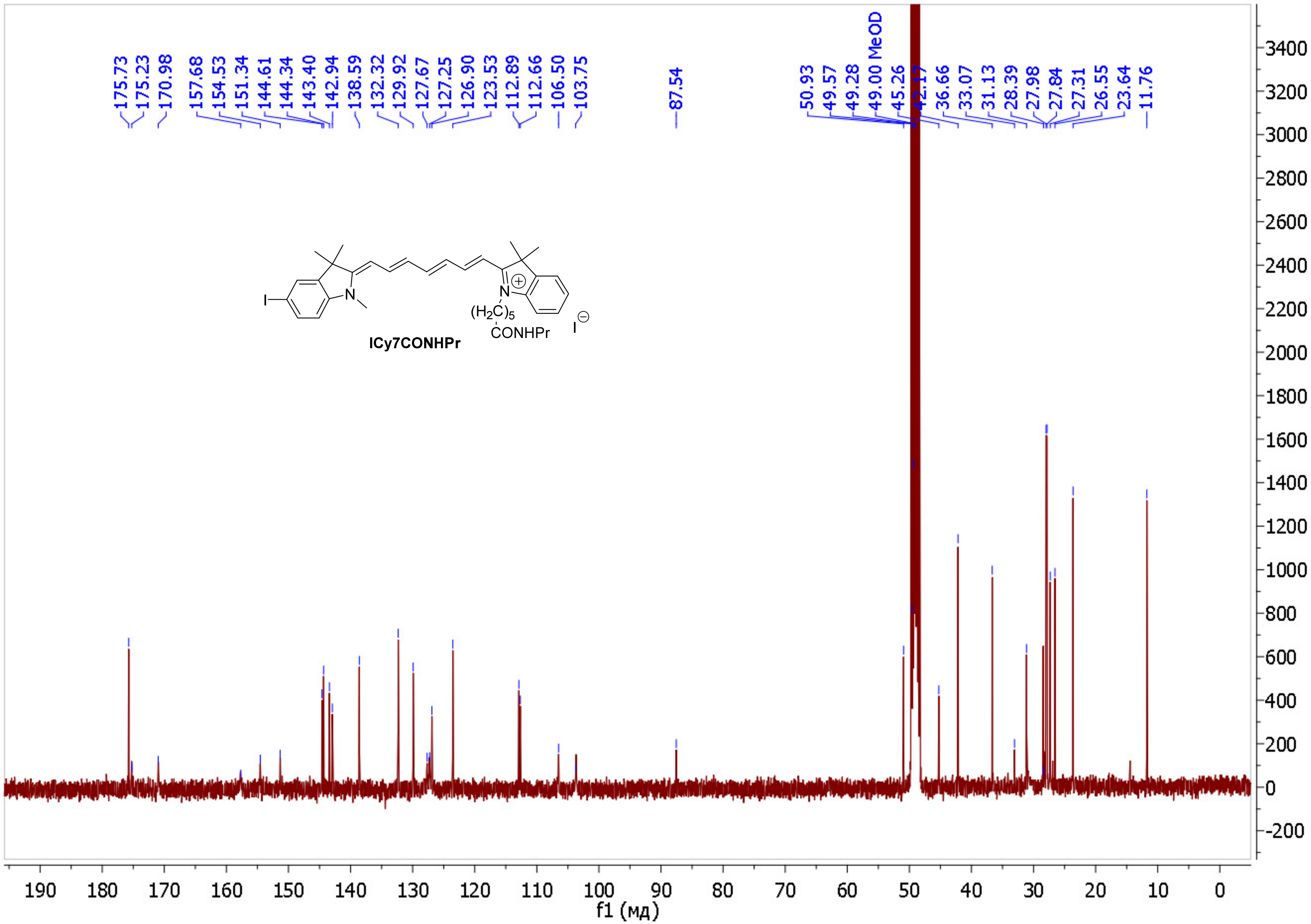

Figure S17. ^{13}C NMR spectrum of **ICy7CONHPr** (100 MHz, CD_3OD).

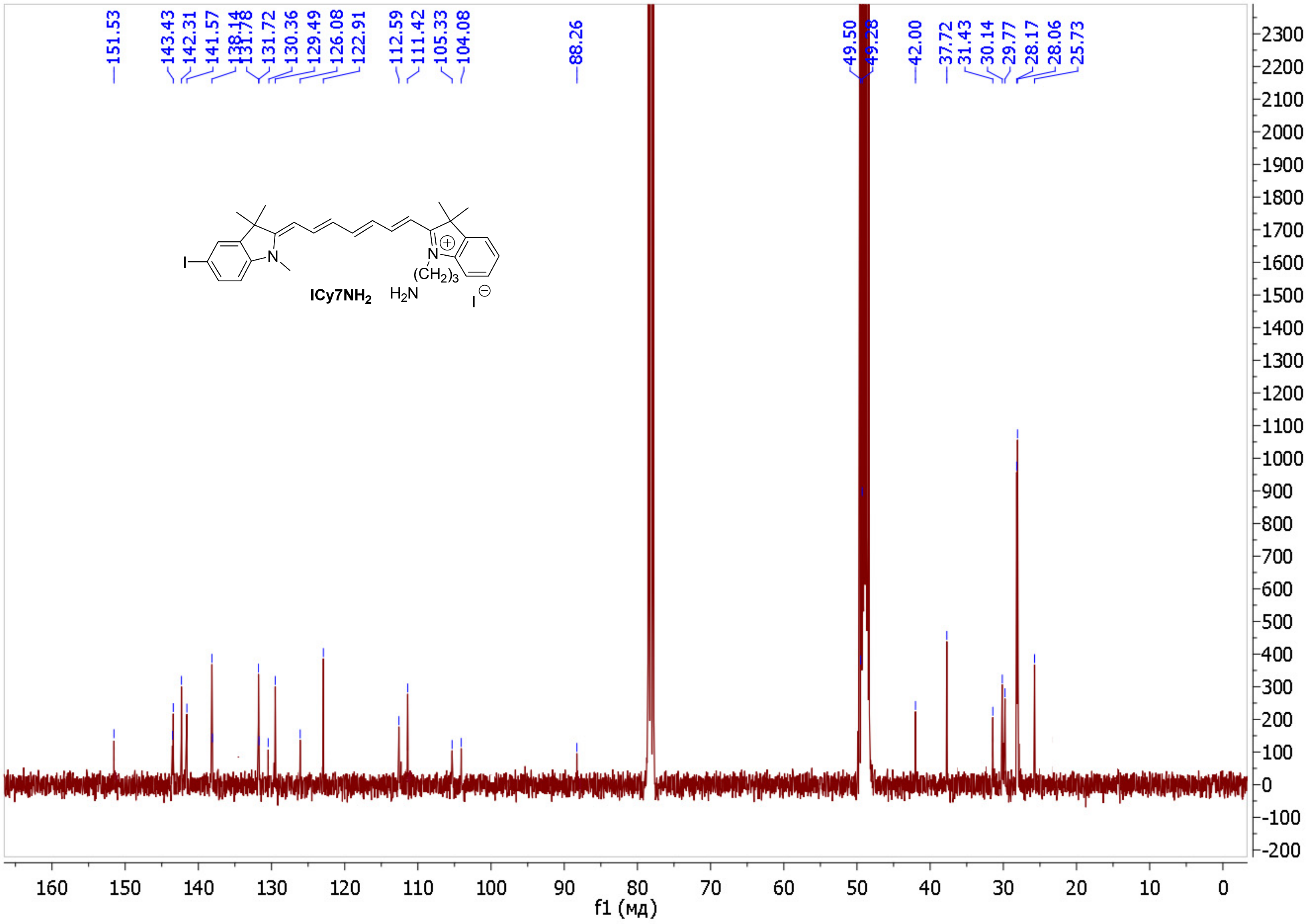

Figure S18. ^{13}C NMR spectrum of **ICy7NH₂** (100 MHz, CD_3OD).

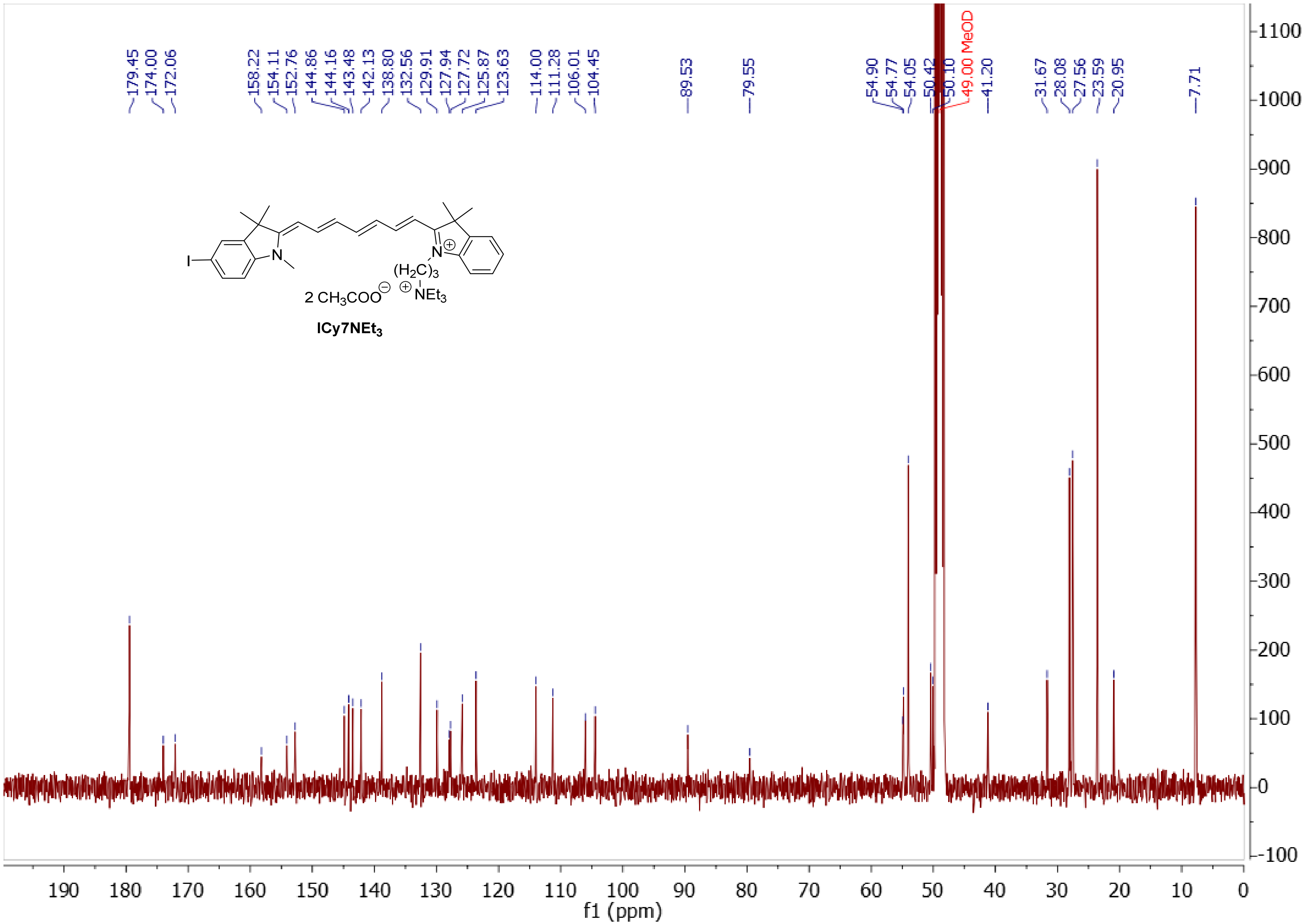

Figure S19. ^{13}C NMR spectrum of **ICy7NEt3** (100 MHz, CD_3OD).

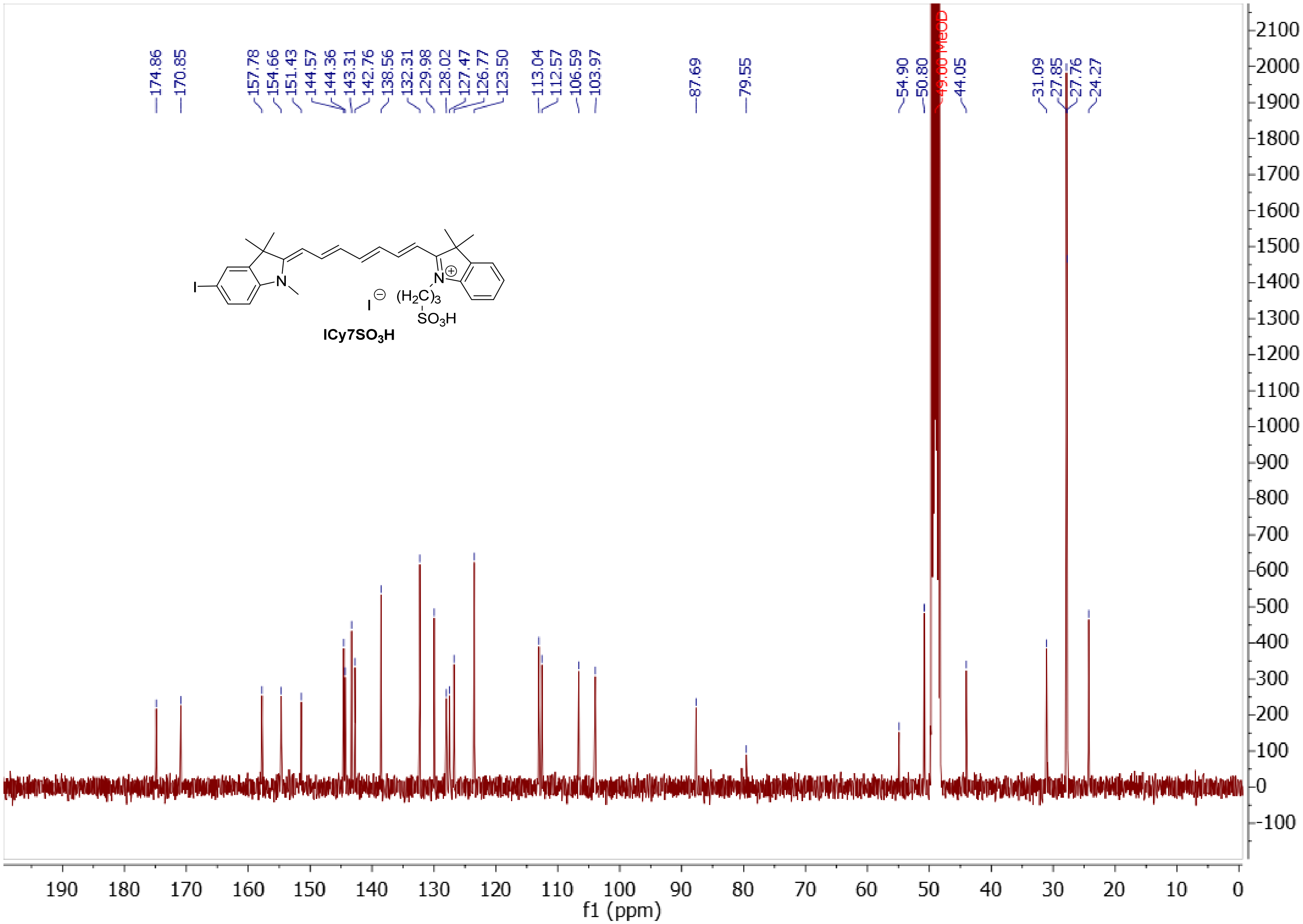

Figure S20. ^{13}C NMR spectrum of **ICy7SO3H** (100 MHz, CD_3OD).

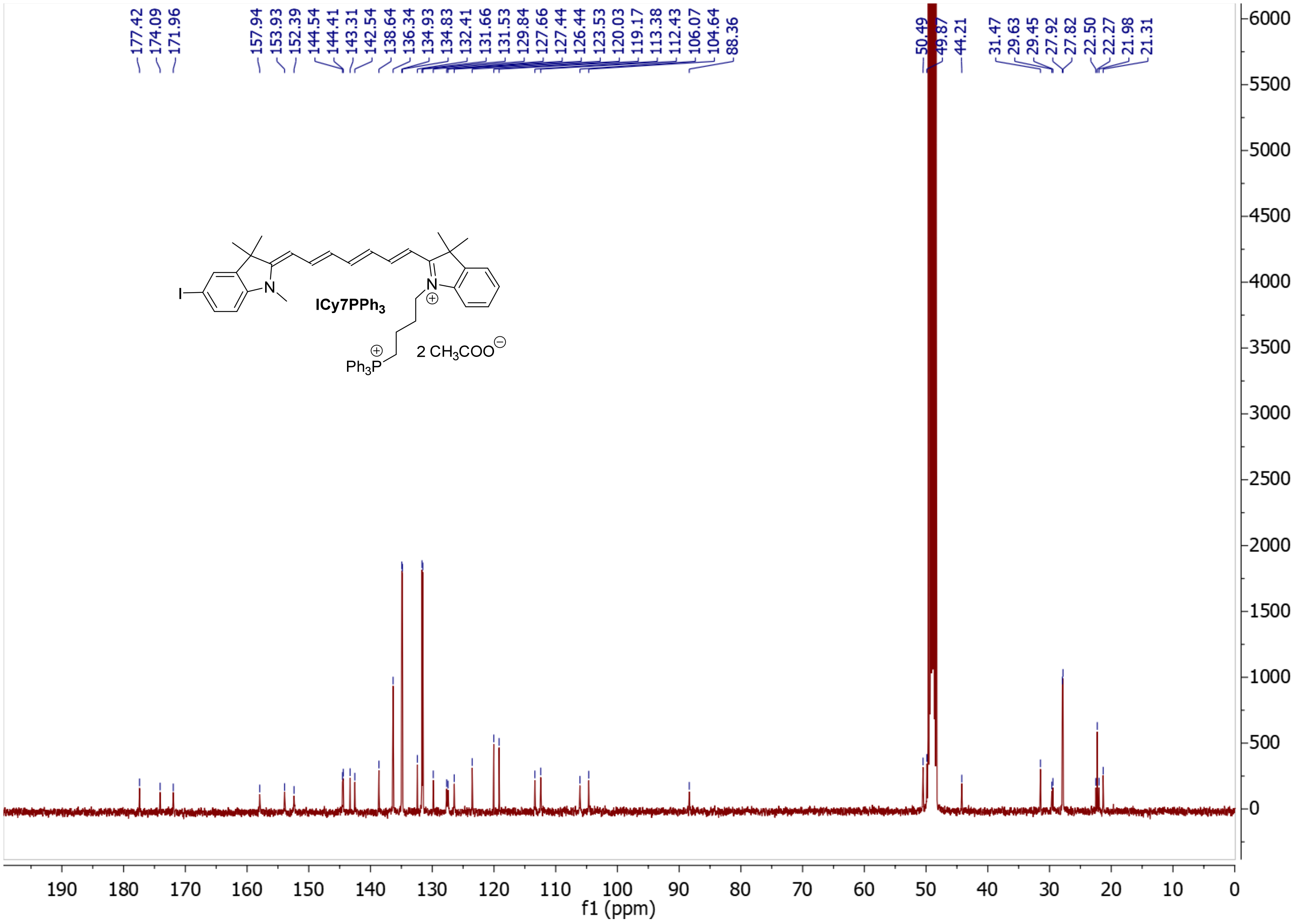

Figure S21. ^{13}C NMR spectrum of **ICy7PPh$_3$** (100 MHz, CD_3OD).

5. HRMS spectra

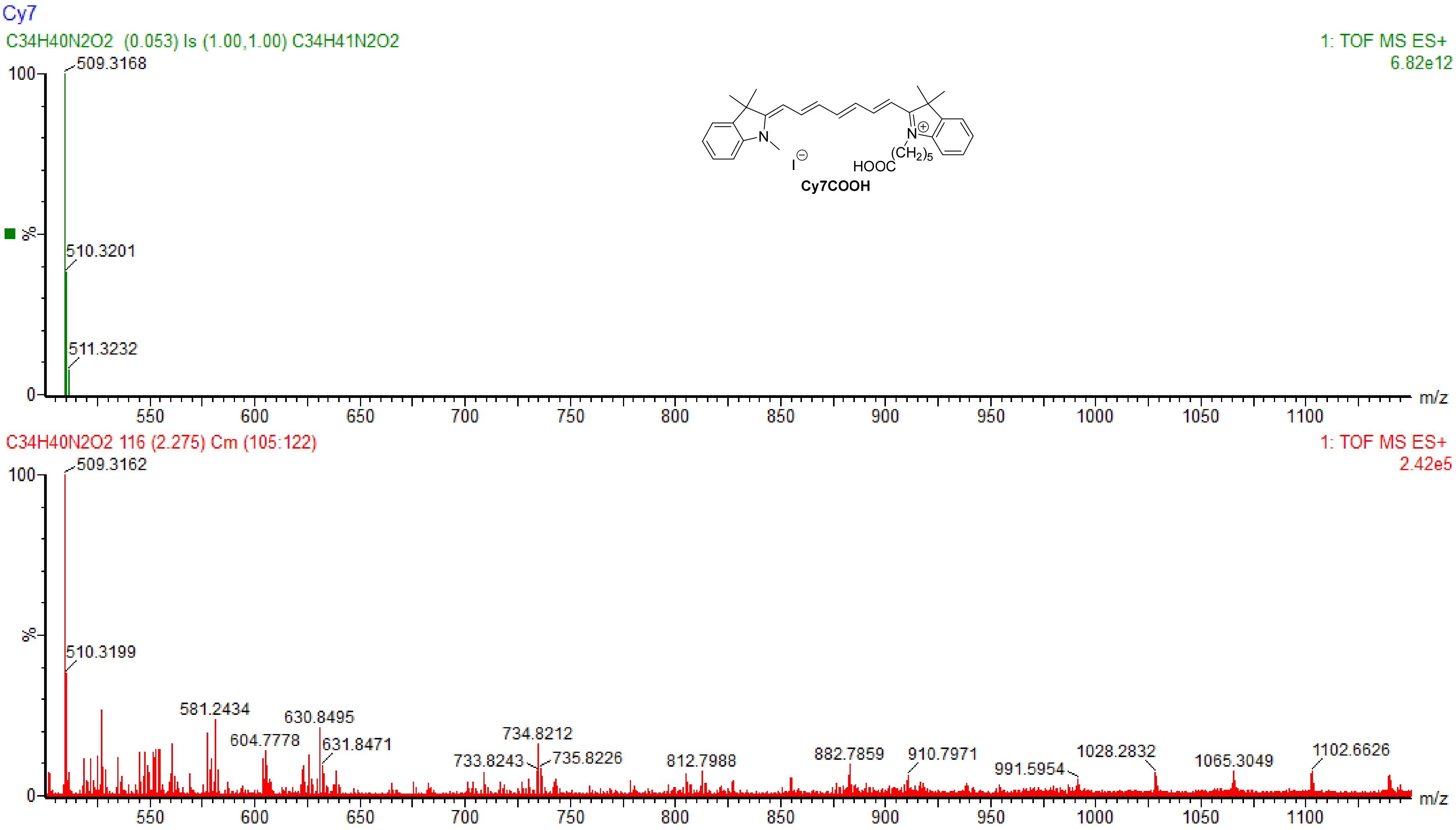

Figure S22. Theoretical (top) and experimental (bottom) HRMS spectrum of **Cy7COOH**.

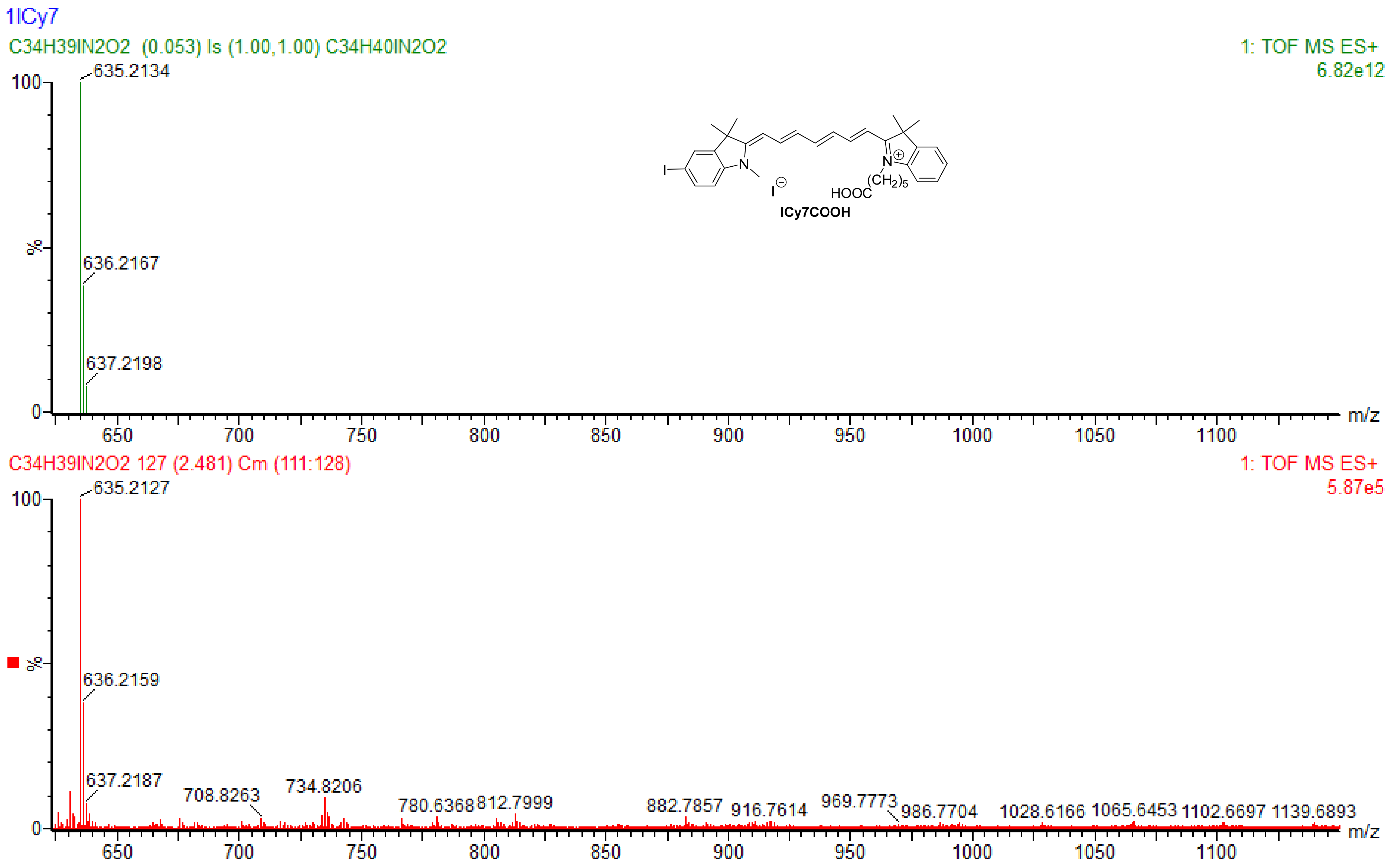

Figure S23. Theoretical (top) and experimental (bottom) HRMS spectrum of **ICy7COOH**.

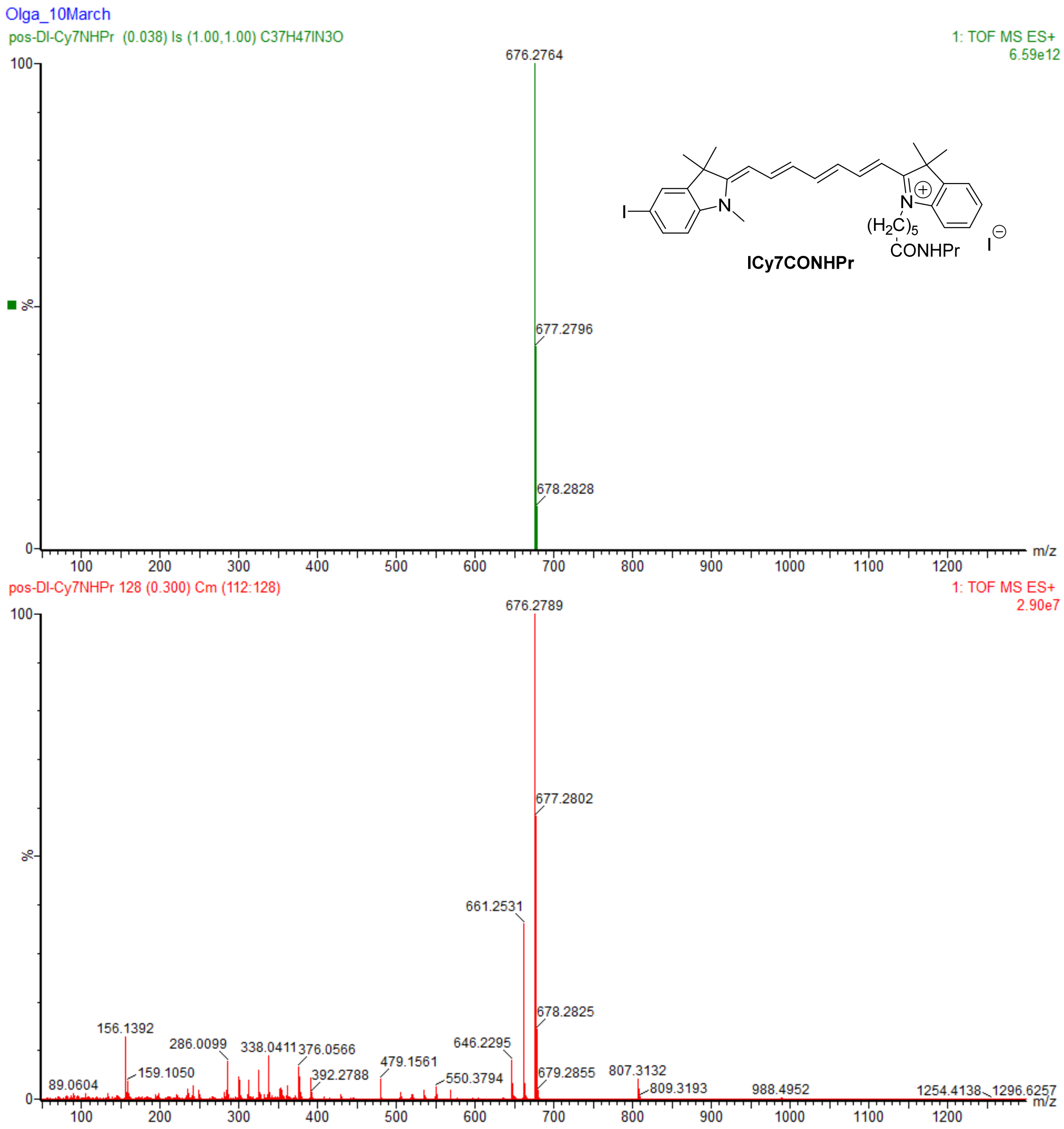

Figure S24. Theoretical (top) and experimental (bottom) HRMS spectrum of **ICy7CONHPr**.

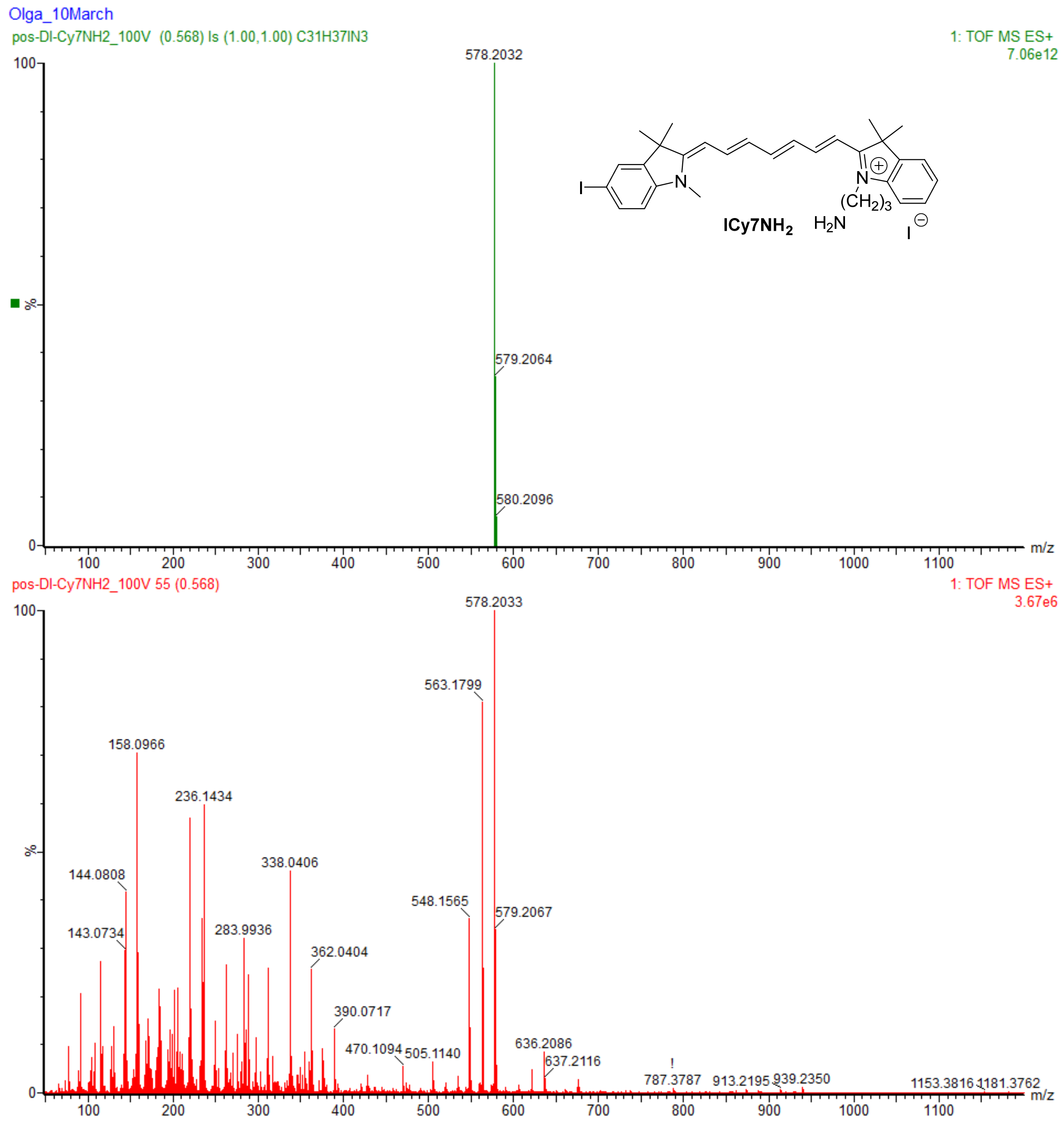

Figure S25. Theoretical (top) and experimental (bottom) HRMS spectrum of **ICy7NH$_2$**.

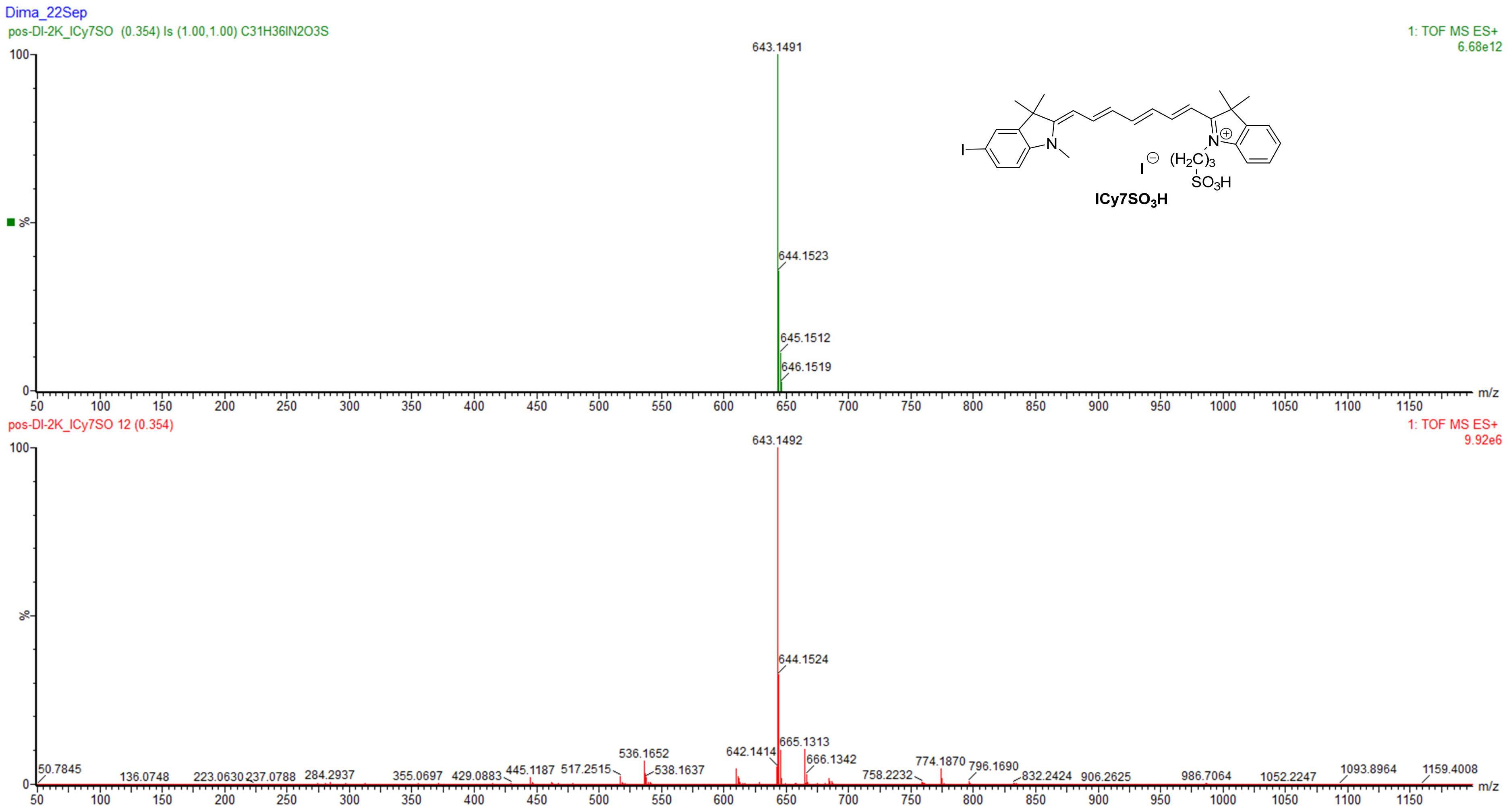

Figure S26. Theoretical (top) and experimental (bottom) HRMS spectrum of **ICy7SO$_3$H**.

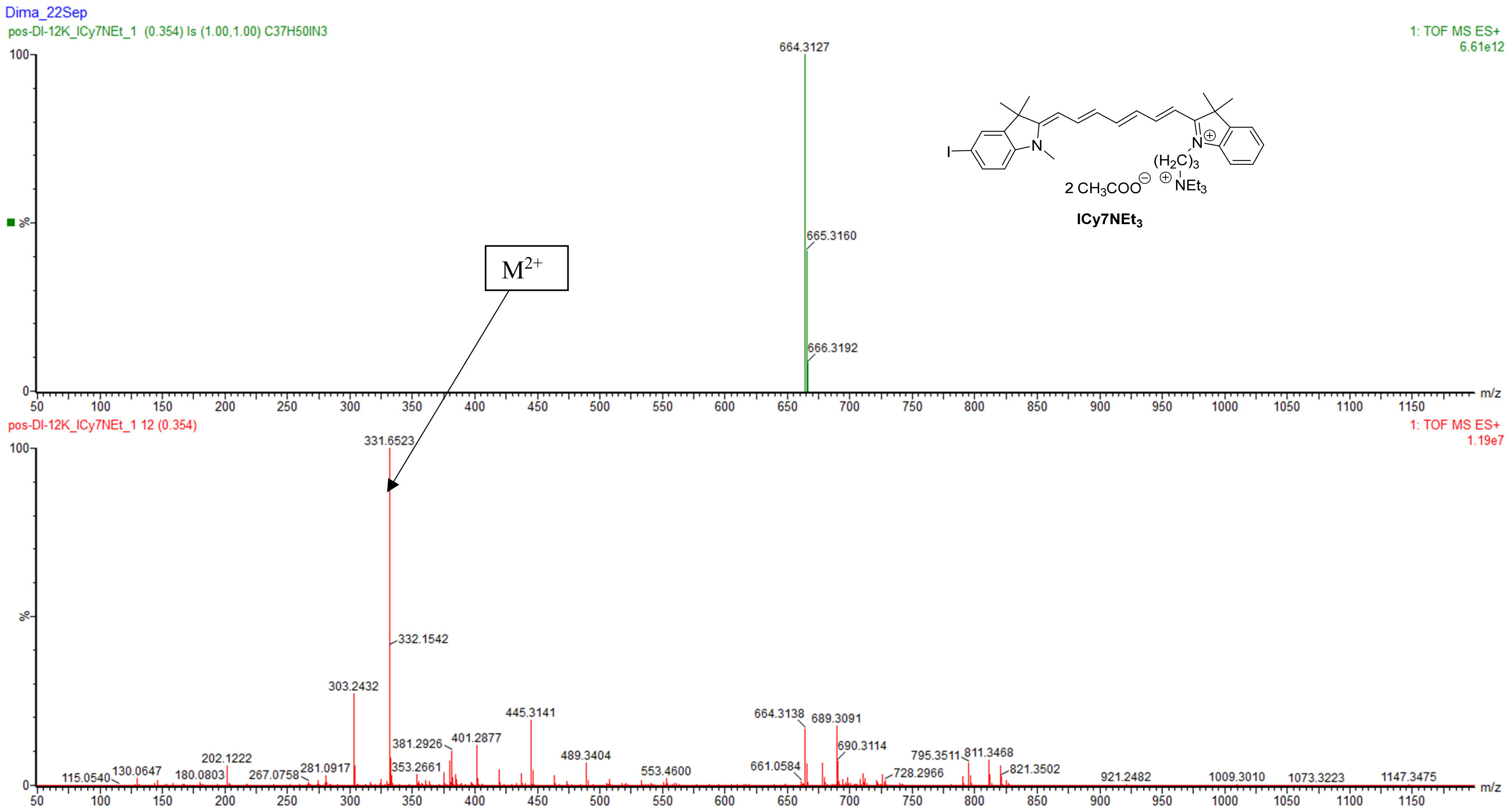

Figure S27. Theoretical (top) and experimental (bottom) HRMS spectrum of **ICy7NEt$_3$**.

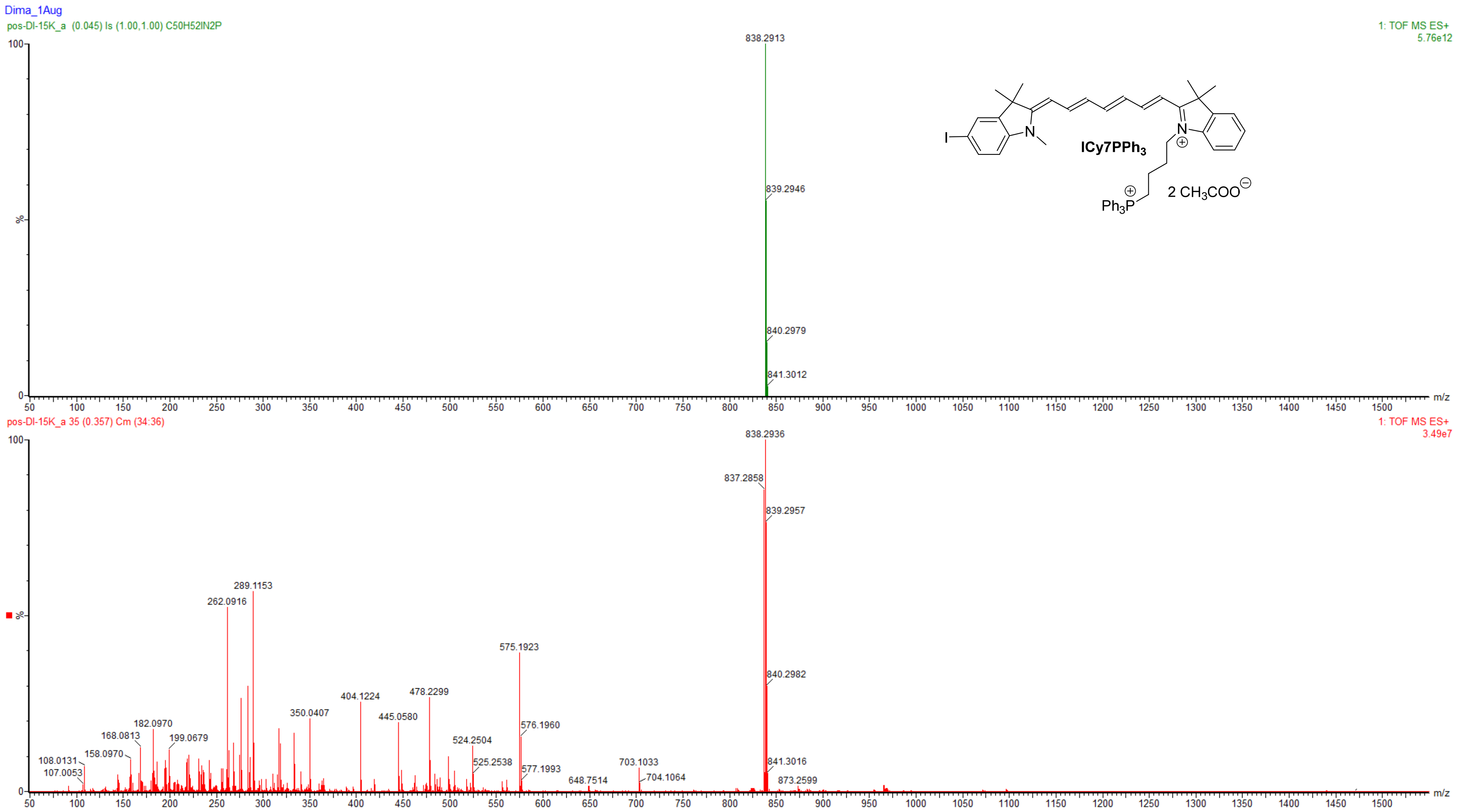

Figure S28. Theoretical (top) and experimental (bottom) HRMS spectrum of **ICy7PPh3**.

6. HPLC data

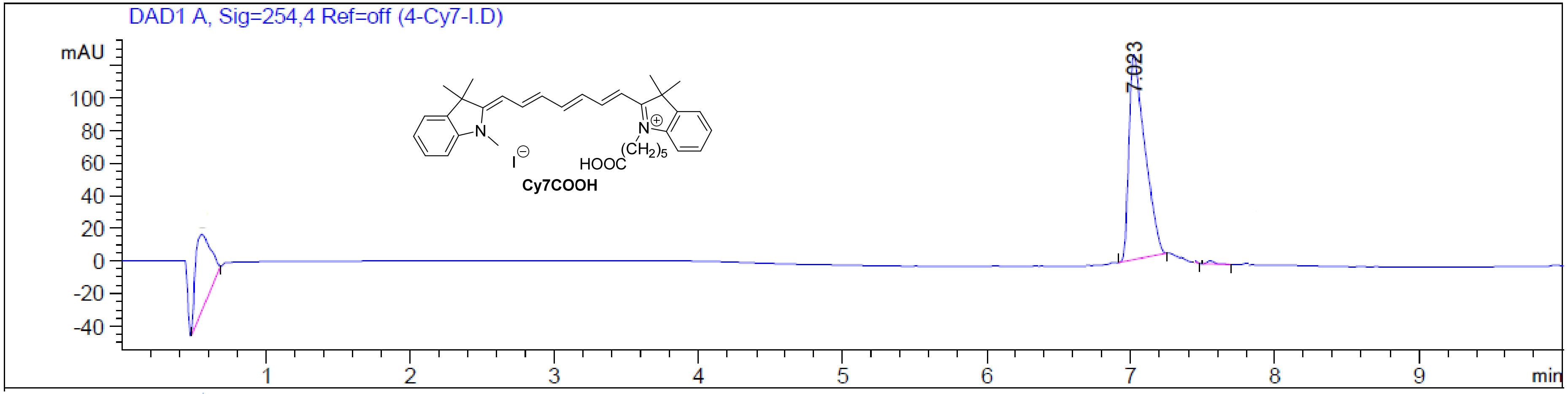

Figure S29. HPLC chromatogram of **Cy7COOH**.

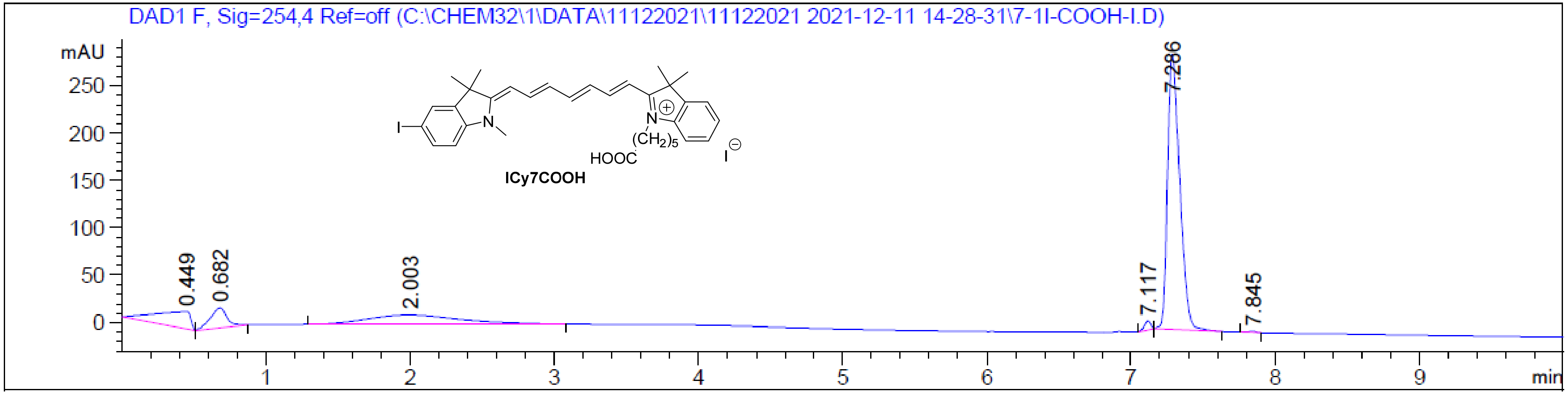

Figure S30. HPLC chromatogram of **ICy7COOH**.

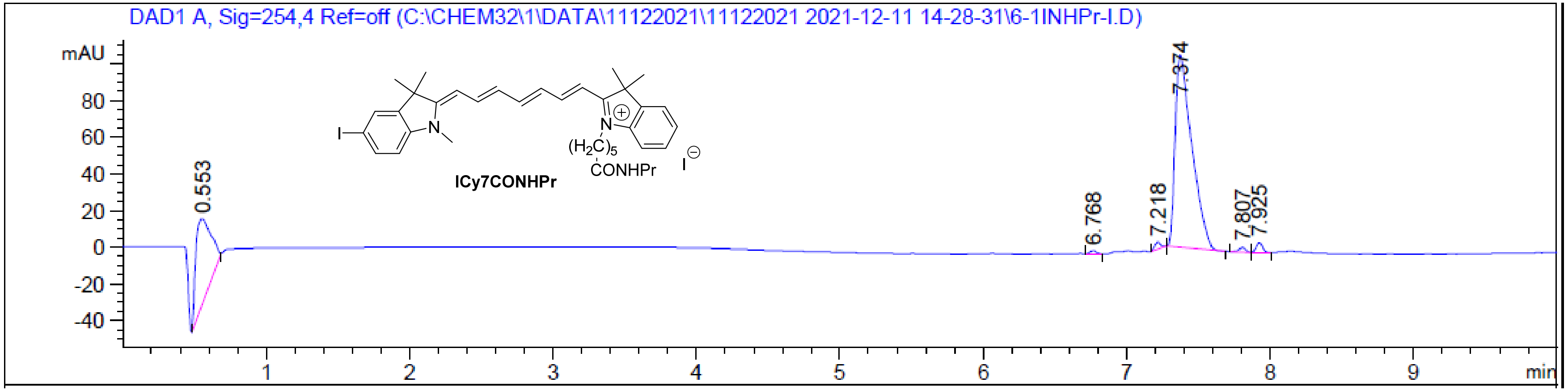

Figure S31. HPLC chromatogram of **ICy7CONHPr**.

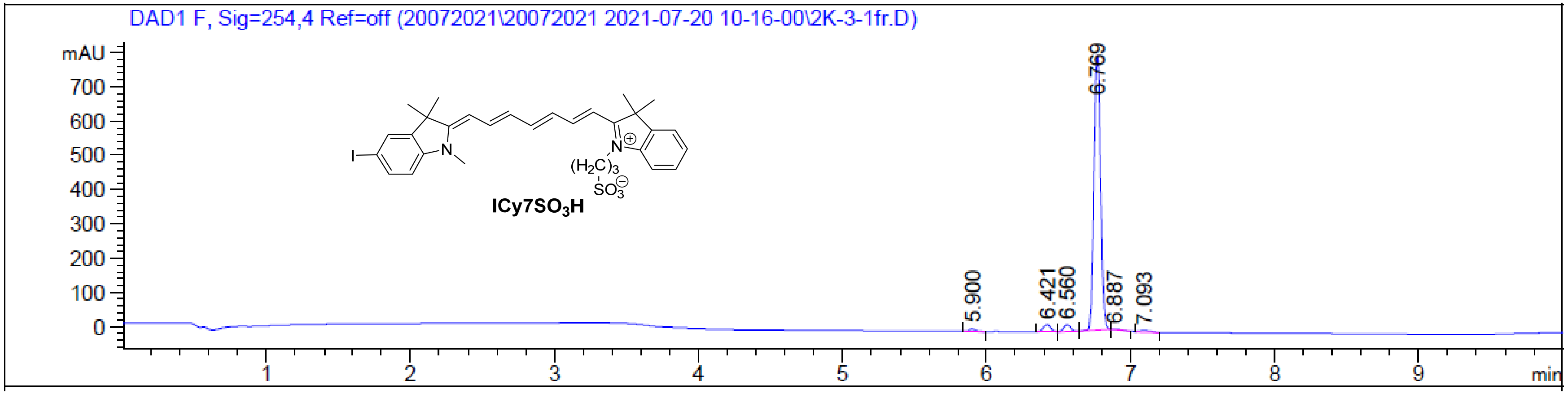

Figure S32. HPLC chromatogram of **ICy7SO$_3$H**.

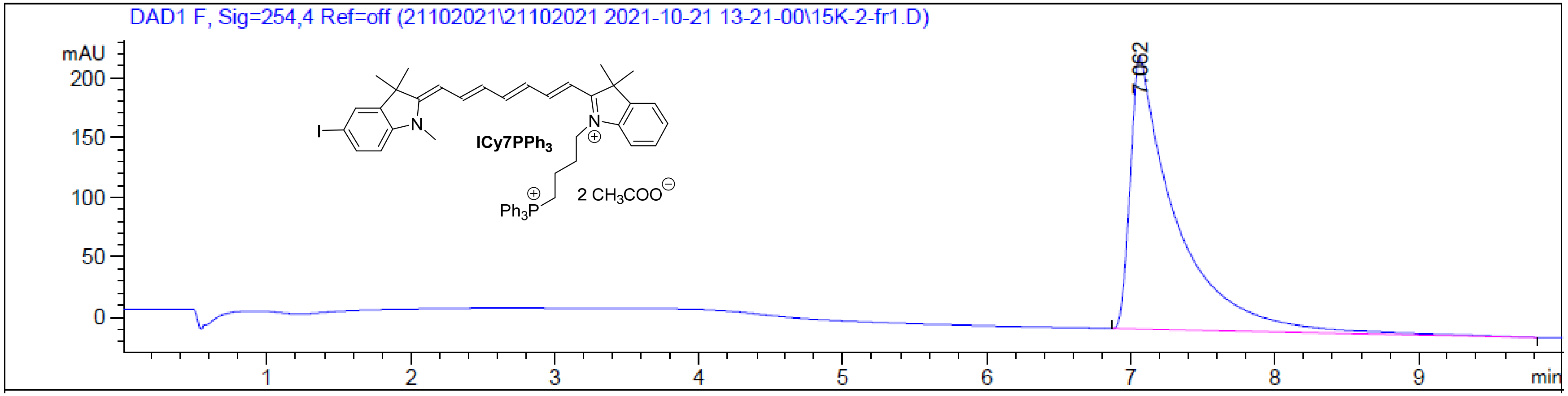

Figure S33. HPLC chromatogram of **ICy7PPh$_3$**.

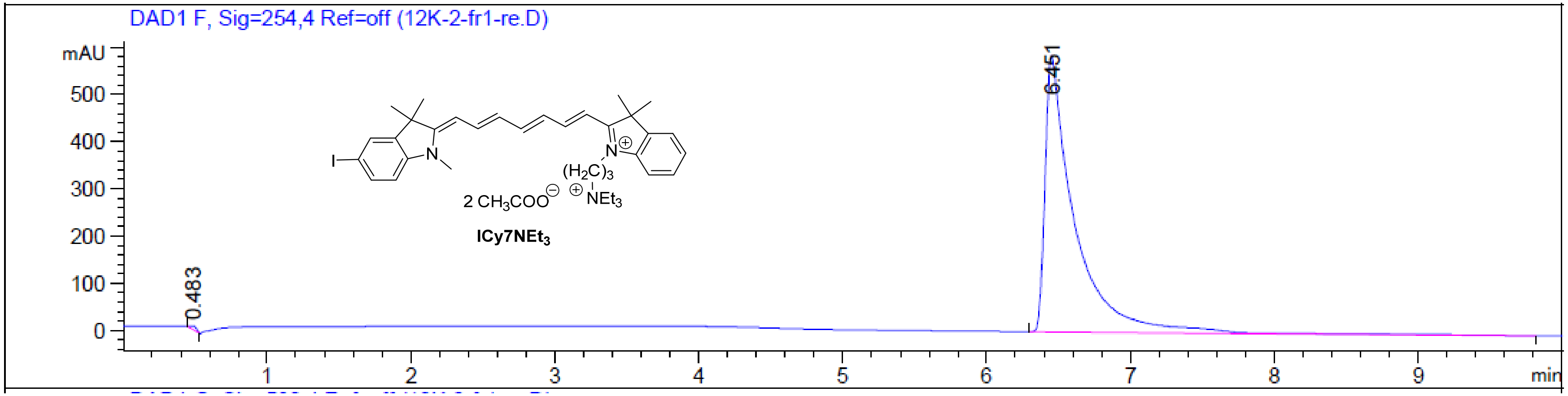

Figure S34. HPLC chromatogram of **ICy7NEt$_3$**.

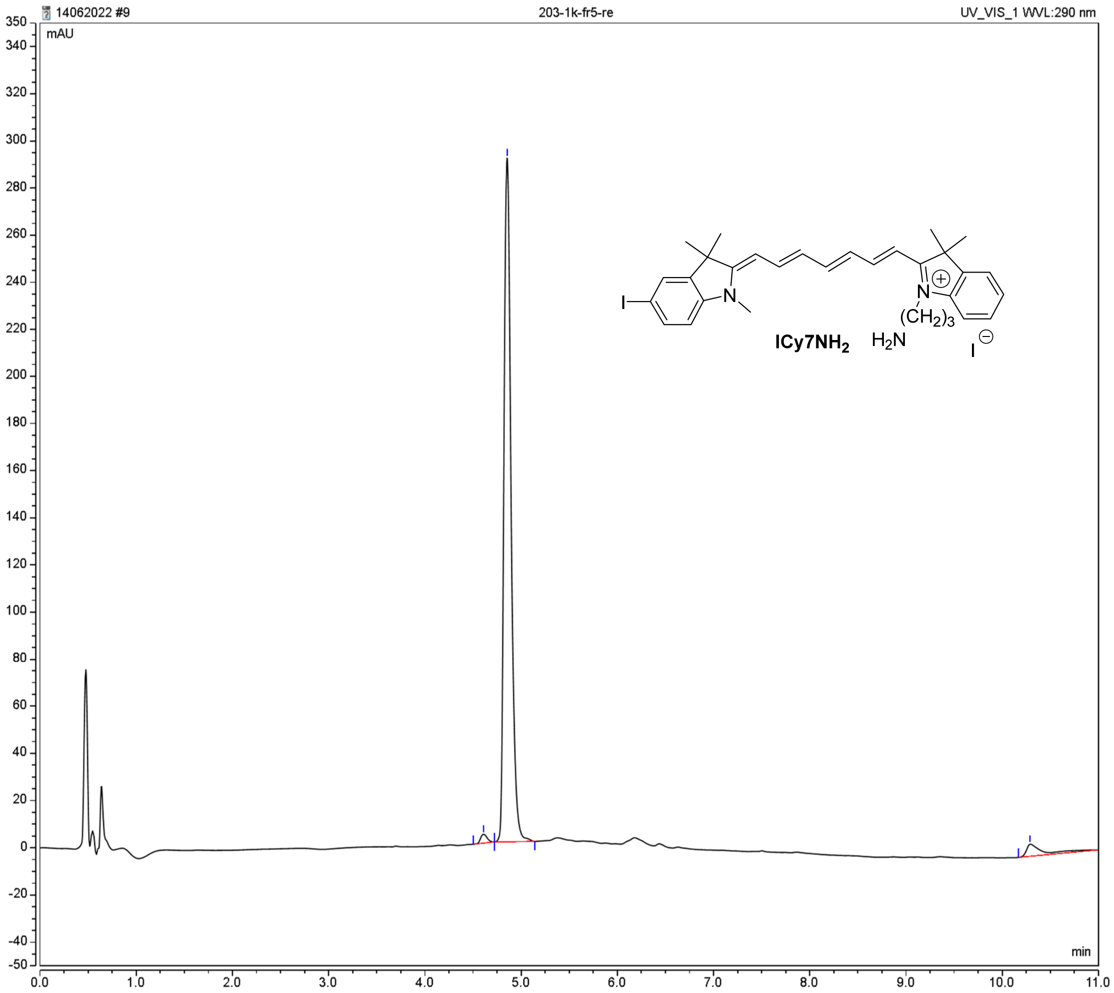

Figure S35. HPLC chromatogram of **ICy7NH₂**.
